# Community-Based Knowledge Translation Strategies for Maternal, Neonatal, and Perinatal Outcomes: A Systematic Review of Quantitative and Qualitative Data

**DOI:** 10.3389/ijph.2023.1605239

**Published:** 2023-04-20

**Authors:** Sandra Milena Montoya-Sanabria, Yesika Tatiana Hernández-Sandoval, Sergio Augusto Cáceres-Maldonado, Diana Catalina Díaz-Barrero, Angélica María Zapata-Matheus, Dauris Lineth Mejia-Pérez, Amaila De La Torre-Arias, Yuldor Eduardo Caballero-Diaz, Catalina González-Uribe, María Teresa Domínguez-Torres, Hong Lien Nguyen, Juan José Yepes-Nuñez

**Affiliations:** ^1^ Institute of Public Health, Pontificia Universidad Javeriana, Bogotá, Colombia; ^2^ School of Medicine, Universidad de los Andes, Bogotá, Colombia; ^3^ Norwegian Institute of Public Health (NIPH), Oslo, Norway; ^4^ Pulmonology Service, Internal Medicine Section, Fundación Santa Fe de Bogotá University Hospital, Bogotá, Colombia

**Keywords:** knowledge translation, maternal health, systematic review, meta-analysis, maternal and child health, neonatal mortality, neonatal health, GRADE approach

## Abstract

**Objective:** To identify and assess the effect of community-based Knowledge Translation Strategies (KTS) on maternal, neonatal, and perinatal outcomes.

**Methods:** We conducted systematic searches in Medline, Embase, CENTRAL, CINAHL, PsycInfo, LILACS, Wholis, Web of Science, ERIC, Jstor, and Epistemonikos. We assessed the certainty of the evidence of the studies using the Grading of Recommendations Assessment, Development, and Evaluation (GRADE) framework.

**Results:** We identified seven quantitative and seven qualitative studies. Quantitative findings suggest that there is a possible effect on reducing maternal mortality (RR 0.65; 95% CI, 0.48–0.87; moderate evidence certainty); neonatal mortality (RR 0.79; 95% CI 0.70–0.90; moderate evidence certainty); and perinatal mortality (RR 0.84; 95% CI 0.77–0.91; moderate evidence certainty) in women exposed to KTS compared to those who received conventional interventions or no intervention at all. Analysis of qualitative studies identified elements that allowed to generate benefit effects in improving maternal, neonatal, and perinatal outcomes.

**Conclusion:** The KTS in maternal, neonatal, and perinatal outcomes might encourage the autonomy of communities despite that the certainty of evidence was moderate.

## Introduction

According to the 2021 Sustainable Development Goals Report, maternal health outcomes have improved. The global maternal mortality ratio (MMR) decreased by 38% between 2000 and 2017, falling from 342 to 211 deaths per 100.000 live births (LBs) (1). However, there were considerable differences depending on the financial level of the nation: 13 fatalities per 100,000 live births in high-income nations, 180 per 100,000 in middle-income nations, and 479 per 100,000 in low-income nations (2). Country-specific MMR varied from three maternal fatalities per 100,000 live births in Finland to 1360 in Sierra Leone, respectively. An MMR of more than 400 maternal deaths per 100,000 live births existed in 24 countries (3). Different social determinants of health can explain these high maternal, neonatal, and perinatal mortality rates. It has been reported that women’s living conditions, particularly poverty, reduce their access to job opportunities or education (4). Similarly, gender-based violence and the existing barriers to access to maternal-child healthcare have resulted in negative maternal, neonatal, and perinatal outcomes (4–6).

Even though decisions to improve healthcare systems are often made based on scientific knowledge, traditional knowledge of indigenous populations has rarely been considered, despite decisions will act on this population (7). Traditional knowledge, and its translation, for healthcare’s benefit, has recently been declared a crucial element in improving maternal, neonatal, and perinatal healthcare (8). Knowledge Translation Strategies (KTS) include a process of understanding between communities and healthcare personnel based on different knowledge and practices, promoting a horizontal interaction between two or more KTS stakeholders (7–10). Thus, implementing KTS may reduce inequalities related to interculturality in healthcare services. For maternal, neonatal, and perinatal health outcomes, this may be a tool to improve healthcare, even superior to conventional vertical strategies historically offered in healthcare systems for the community (8).

Therefore, this study aims to identify the effect of KTS with horizontal engagement between the local community and organizations or programs, in bidirectional communication, on maternal, neonatal, and perinatal outcomes in community healthcare settings.

## Methods

### Search Strategy and Selection Criteria

We followed the PRISMA 2020 (11) guideline for reporting the findings of this systematic review (SR) (Supplementary Material S1). We included quantitative and qualitative studies that evaluate KTS compared with standard care or no intervention to improve maternal, neonatal, and perinatal outcomes. We incorporated the KTS definition provided by the Canadian Institutes of Health Research: “a dynamic and iterative process that includes the synthesis, dissemination, exchange and ethically sound application of knowledge to improve health, provide more effective health services and products, and strengthen the healthcare system” (12). We chose this definition because it implies a process of knowledge exchange and understanding knowledge between two populations in a bidirectional interaction. We included randomized and non-randomized studies (cohort studies, case-control studies, before-and-after studies, cross-sectional studies, and case-series studies) and qualitative studies [case studies, participatory action research (PAR) studies, and grounded theory studies].

### Outcomes

We included patient-important outcomes in our SR. For quantitative studies, we focused on maternal morbidity, maternal mortality, mothers’ satisfaction with caregiving, maternal mental health disorders, spontaneous vaginal delivery, caesarean delivery, instrumental vaginal delivery, intact perineum, initiation of breastfeeding, neonatal mortality, neonatal morbidity, perinatal mortality, and community impact. We did not pre-specify outcomes for the qualitative studies. Outcome definitions are reported in the Supplementary Material S2.

Electronic databases and journal repositories [Medline, Embase, CINAHL, PsycInfo, Lilacs, Wholis, Web of Science, ERIC, PASCASL IPA, and Jstor (*via* Ovid)] were searched from inceptions to June 2022. We designed the search strategies with the support of an expert librarian, and the final search strategies were peer reviewed by a second librarian. We did not restrict by language. We searched the reference lists of retrieved studies and contacted authors and experts in the field. We also reviewed databases of the ministries of health of countries in Latin America and Africa, UN agencies, OpenGrey, WONDER, OPS IRIS, Epistemonikos, and documents published by midwife groups and/or professional associations of certified midwives. We previously published the protocol in Open Science Framework: https://osf.io/8u532/. Search strategies are reported in the Supplementary Material S3.

### Data Collection

Two reviewers, independently and in duplicate, screened the records by title, abstract, and full text. We independently and in duplicate extracted the data from the studies that met our inclusion criteria. We used Covidence^®^ (13) and CADIMA (https://www.cadima.info/index.php) for the screening process and pre-piloted forms for data extraction. Before each screening process, we assessed the level of agreement between reviewers using Cohen’s kappa coefficient (14). We resolved disagreements by consensus with a third reviewer. We extracted the following data for each individual study: study identifier; study design; setting; population characteristics; quantitative outcomes; source of funding; intervention and comparator; and study limitations.

### Data Analysis

#### Evaluating Risk of Bias in Individual Studies

Four reviewers (SC, DD, YH, AZ), working in duplicate and independently, assessed the risk of bias for each quantitative and qualitative study. Disagreements were solved by discussion, and, in case a consensus was not reached, a third reviewer helped to solve the conflict (SM, JY-N). We determined risk of bias in randomized studies using the Cochrane Risk of Bias (RoB) Assessment Tool 2.0 (15). For a specific outcome, we considered a study as *high risk of bias* if the study was judged to be at high risk of bias in at least one domain or if a study had some concerns for multiple domains. For observational studies, we used the Cochrane Risk of Bias assessment tool for non-randomized studies (ROBINS-I) (16). Per outcome, we considered a study to be at *serious risk of bias* if the study was judged to be at serious risk of bias in at least one domain but not at critical risk of bias in any domain. For qualitative studies, we assessed the evidence critically using a CASP (Critically Appraisal Skills Program) checklist (17).

#### Quantitative Data Analyses

We calculated relative risks (RR) and 95% confidence intervals (95% CI). We determined heterogeneity among individual studies by visually inspecting forest plots and using the Q statistic and the I^2^ index (18). We obtained relative risk calculations using a random effects model since we expected heterogeneity among individual studies. We used the Review Manager 5.4.1 software Field (19) for statistical analyses. To explore heterogeneity, the following subgroups were prespecified: 1) maternal morbidity, 2) mental health disorder, 3) route of delivery, 4) neonatal mortality, 5) neonatal morbidity, and 6) other morbidities. We planned a sensitivity analysis to evaluate the robustness of effects calculations by assessing a random effects model compared to a fixed effects model.

Information biases (such as publication bias) were explored using funnel plots where there were ten or more studies in the meta-analysis (19, 20). We assessed funnel plot asymmetry visually. In case of asymmetry, we performed exploratory analyses to investigate it.

#### Qualitative Data Analyses

In the case of studies using qualitative methods, an analytical approach adapted from grounded theory methods has been followed to extract and analyze qualitative data (meta-synthesis) (21, 22). Based on a constant data comparison, this inductive analytical technique fits our objective of adding qualitative evidence and provides new conceptual interpretations integrating findings across studies (22). We conducted an open interpretative coding process to generate synthetized interpretative codes and subcategories. Subsequently, following an axial and selective coding process, we generated meta-categories for qualitative synthesis. We used the NVivo Version 12 software for the qualitative synthesis analysis (23).

#### Certainty of the Body of Evidence

For quantitative studies, we evaluated the certainty of the evidence for each outcome using the Grading of Recommendations, Assessment, Development and Evaluations (GRADE) framework as high, moderate, low, or very low (24). For qualitative studies, we evaluated the degree of confidence in findings using the GRADE-CERQual tool (25). The domains can be graded as having high confidence, moderate confidence, low confidence, and very low confidence. We used the electronic tool GRADEpro GDT (https://gradepro.org) to create an Evidence Profile and a Summary of Findings tables (26). We summarized qualitative findings from the coding and categorization process using the GRADE-CERQual interactive Summary of Qualitative Findings tool: https://isoq.epistemonikos.org.

## Results

**FIGURE 1:**
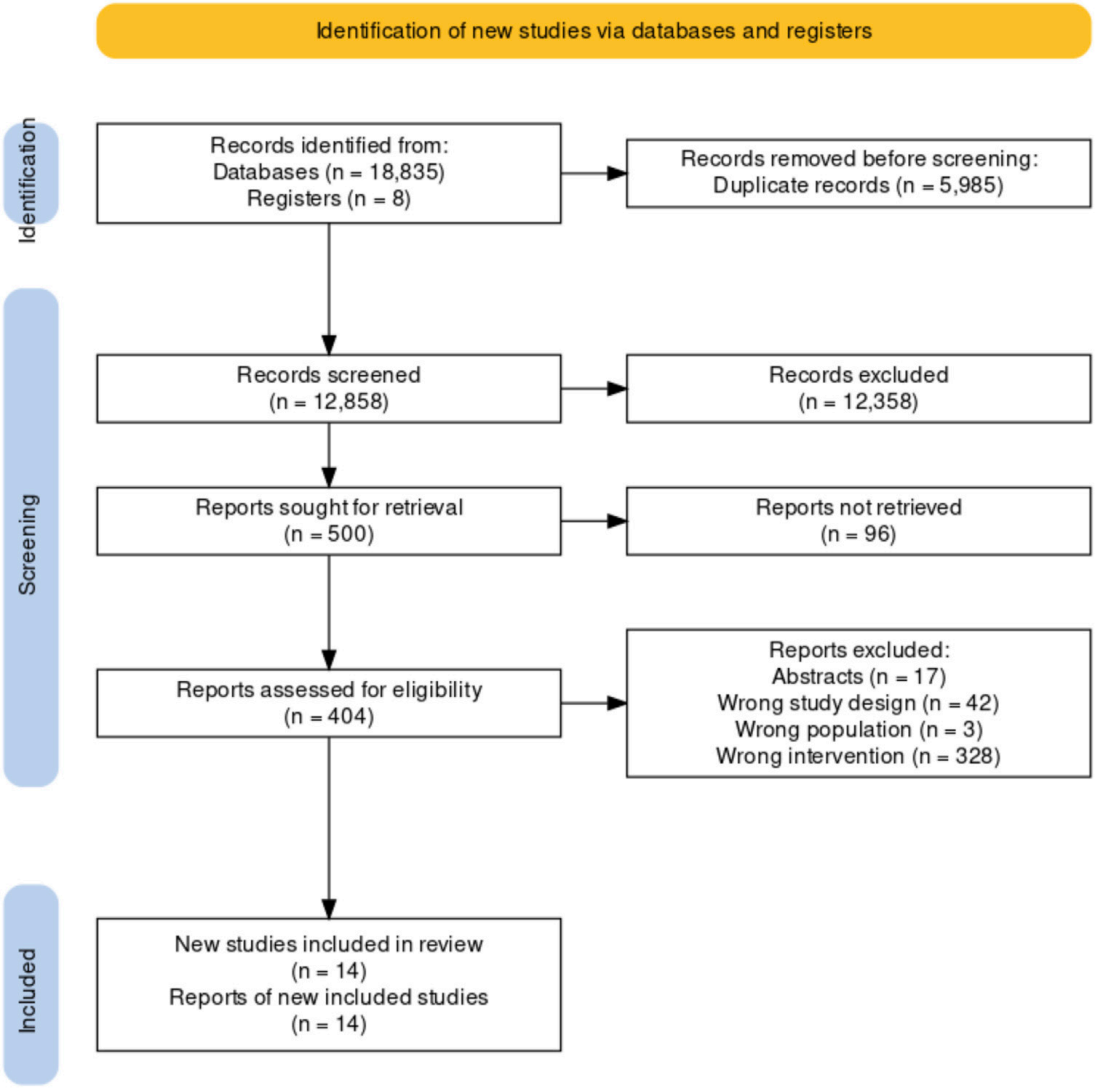
PRISMA 2020 flow diagram (Bogota, Colombia. 2023).

We retrieved 12,850 unique records from our database search and selected 501 records according to title and abstract screening. The full text of ninety-six registries could not be retrieved even after contacting their authors. A total of twelve reports (27–38) of the 405 reviewed in full text met our inclusion criteria. We identified two additional studies while reviewing the referent list of two SRs captured for our search strategy (39, 40). We included 14 studies reported in 14 records. Overall, we included seven quantitative studies (28, 33–37, 39), five randomized studies (28, 34–37) and two non-randomized studies (33, 39); and seven qualitative studies (18, 27, 29, 31, 32, 38, 40). Qualitative studies included a variety of methodologies such as participatory action research (*n* = 5, 71.4%) (27, 29, 31, 38, 40), qualitative case study (*n* = 1, 14.3%) (39) grounded theory (*n* = 1, 14.3%) (32), and a mixed-approach study (*n* = 1, 14.3%) (30) that conducted a content analysis. We included the qualitative approach of the mixed study because of its relevance to our systematic review. Figure 1 shows a summary of the selection process (41). Principal characteristics of the studies that fulfilled our inclusion criteria are shown in Table 1.

**TABLE 1:** Characteristics of the studies included (Bogota, Colombia. 2023).

**Study**	**Country**	**Type of study**	**Number of participants**	**Population**	**Intervention**	**Comparator**	**Follow-up period**	**Outcomes**
**Quantitative studies**									
Colbourn, 2013 (28)	Malawi	Cluster randomized controlled trial	Sixty-two clusters, approx. 4,000 persons per cluster	Pregnant women from three districts of Central Malawi	Community mobilization	No intervention	Intervention period: 27 months	Maternal, neonatal, and perinatal mortality	
Maldonado, 2020 (33)	Kenya	Cohort study	326 participants	Pregnant women attending their first antenatal care visit at an health facility in Bunyala, Kenya	Configuration of women’s groups	Standard of care	Intervention period: 12 months	Neonatal and maternal mortality	
Manandha, 2004 (34)	Nepal	Cluster randomized controlled trial	Twenty-four clusters, approx. 7,000 persons per cluster	Married women of childbearing age between 15 and 49 years of age from the district of Makwanpur, Nepal	Configuration of women’s groups	Improvements in health facilities	Intervention period: 24 months	Maternal and neonatal mortality	
Morrison, 2020 (35)	Nepal	Cluster randomized controlled trial	Family size was a median 7 (7–8) in both groups	Women aged 12–49 years who delivered infants	Participatory action and learning cycles	No intervention	Intervention period: 23 months	Neonatal mortality	
Rahman 2019 (39)	Bangladesh	Quasi-experimental study	737 participants.	Married women between 15 and 49 years of age with an obstetric history of previous from the district of Netrokona, Bangladesh	Participatory interventions packages	No intervention	Intervention period: 24 months	Community impact	
Tripathy 2010 (36)	India	Cluster randomized controlled trial	Thirty-six clusters, 18,775 persons in total	Women between 15 and 49 years of age who gave birth during the study in Jharkhand and Odisha, India	Participatory action and learning cycles	Meetings to discuss the management of local health services	Intervention period: 36 months	Maternal, neonatal, and perinatal mortality	
Tripathy 2016 (37)	India	Cluster randomized controlled trial	Thirty clusters, approx. 5,000 persons per cluster	Women of childbearing age (15–49 years of age) from five rural districts of Jharkhand and Odisha, India	Participatory action and learning cycles	Improvements in the community’s health, sanitization, and nutrition committees	Intervention period: 24 months	Maternal and neonatal mortality	
**Qualitative studies**									
Alcock, 2009 (27)	India	Participatory Action Research (PAR)	Approx. 180 participants	Female peer facilitators from twenty-four neighborhoods in vulnerable conditions of six municipalities in Mumbai, India	Participatory action cycles	No comparator	Intervention period: Not reported. Started in 2006	Three emergent topics: Routine activities, perception of the *sakhis*’ role and their credibility.	
Esienumoh, 2018 (29)	Nigeria	Participatory Action Research (PAR)	1 PAR group of twelve people who represented every stakeholder	Women between 26 and 35 years of age from a rural community at South Nigeria	Configuration of Action-Participation groups	No comparator	Intervention period: 24 months	Six main topics: Ignorance, maternal health problems, socio-cultural factors, birth practices, poverty, and physical-environmental factors	
Higgins-Steele, 2015 (30)	Sierra Leone	Qualitative findings (content analysis)	297 participants	Participants from the district of Kailahun in the eastern province of Sierra Leone	Quality circles	No comparator	Intervention period: 24 months	Organizational skills and self-reported perceptions on relationships	
Lapierre, 2005 (32)	Canada	Grounded Theory	Not explicit	A group of pregnant women living in poverty, a group of mothers from the community, and a group of health professionals	Emancipation processes	No comparator	10 months for group activities and data collection	It strengthened the self-confidence, empowerment, trust, and the capacity to learn and listen of pregnant women	
Joseph, 2021 (31)	Tanzania	Qualitative case study	Eighty-six participants	Participants in this study were purposively sampled from those who directly participated in the selected wards in Kilolo and Mufndi districts.	Configuration of women’s groups	No comparator	No reported	Community readiness to adopt the interventions, the role of community leaders, stakeholders’ engagement, and support of local health systems	
Sarmiento, 2020 (38)	Mexico	Participatory research	326 participants	Four Indigenous municipalities with access to usual healthcare	Participatory intervention packages	No comparator	Intervention period: 12 months	Pregnancy complications	
Rath, 2010 (40)	India	Participatory Action Research (PAR)	Eighteen clusters, 244 groups of women	Participants from three bordering districts of Jharkhand (West Singhbhum and Saraikela Kharsawan), and Orissa (Keonjhar) states in India	Participatory action and learning cycles	No comparator	Intervention period: 36 months	Six emergent topics that influenced the impact of the intervention: Acceptability, Participatory approach for the development of knowledge, skills and critical awareness, community inclusion, active recruitment of newly pregnant women	

In total, approximately 53,521 participants were included in all studies. The quantitative studies involved 52,579 participants, and 942 patients were included in the qualitative studies. Thirteen of the studies (27–31, 33–40) were developed in rural districts and/or locations with low socioeconomic conditions, and the remaining study was conducted in an urban area (32). We identified eight KTS in the fourteen studies included in our review: 1) community mobilization (28); 2) configuration of women’s groups (31, 33, 34); 3) participatory action and learning cycles (35–37, 40); 4) participatory intervention packages (38, 39); 5) configuration of Action-Participation groups (29); 6) participatory action cycles (27); 7) quality circles (30); and 8) emancipation processes (32). Further details of each strategy are described in Table 2.

**TABLE 2:** Knowledge translation strategies (Bogota, Colombia. 2023).

Strategy/Context	Strategy
**Community mobilization**	
Carried out in three districts of Malawi’s Central Region with limited access to health services, electricity, sanitization services, and high illiteracy (28).	Participatory groups of women through a methodology of action cycles in four phases, identified and prioritized maternal health issues, planed solutions at a local level and took actions to execute them, implemented the action plan, and evaluated the actions.
**Configuration of women’s groups**	
Kenyan women attended group health education and microfinance sessions; each session consisted of a 60–90-min participatory lesson (33).	Strategies for knowledge exchange used an illustrated flipchart with an accompanying discussion guide. Each group also delineated personal goals they wished to accomplish during the program.
Strategy carried out in 43 villages in Nepal; 94% of births occurred out of health facilities and illiteracy percentage among women was up to 57% (34).	Participatory groups of women were configured to work in two phases: 1) main maternal health problems were identified and prioritized, and solutions to these problems were planned, and 2) solutions proposed were implemented and evaluated.
Study conducted in Tanzania. The districts exhibited low antenatal care uptake (31).	Participatory Action Research was implemented through two phases: 1) covered identification of antenatal care problems, and 2) involved developing strategies to address the prioritized antenatal care problems.
**Participatory action and learning cycles**	
Study conducted in India, in rural districts where the percentage of illiteracy among women was up to 65%, and with limited access to health services (37).	Groups of women discuss maternal health problems in meetings in four phases: 1) identified y and prioritized maternal health problems by using image cards and games, 2) groups listened to stories that talked about the causes of problems and potential solutions, 3) groups implemented solutions, and 4) groups evaluated the overall process.
Studies conducted in India. Districts located in rural regions with limited access to health services and 80% of births occurred out of health facilities (36, 40).	In one study, groups of women were configured to discuss problems related to pregnancy, birth, and the post-natal period, and then action cycles were carried out (40). The other study implemented three strategies in groups: picture-card games, role play, and storytelling, to help discuss the causes and effects of problems in mothers and infants (36).
Study reported a four-day workshops with Health Management Committees to improve their capacity for planning and action and supported female community health volunteers to run women’s groups (35).	Women’s group intervention was trained in facilitation skills, participatory learning, and action cycle process. They discussed barriers to institutional delivery and ways to address them. Then, organized community groups examined support for strategy implementation. After the strategies were implemented, the women’s group reflected on their progress and planned and implemented further strategies.
**Participatory intervention packages**	
Study conducted in Mexico with Indigenous communities to help them address poor maternal health (38).	Dialogue between indigenous community and researchers was conducted in three steps: 1) trust building and partnership based on mutual respect and principles of cultural safety, 2) to listen and to adjust the lexicon, and 3) codesign, evaluation and discussion to identify benefits of supporting traditional midwifery on maternal outcomes.
Study conducted in Bangladesh in a village with scarce economical resources. The health services coverage for maternal health was limited and the percentage of illiteracy was nearly 60% (39).	Processes of knowledge exchange created intervention packages focused on four areas: 1) warning signs during pregnancy, 2) knowledge about maternal health rights, 3) preparation for childbirth and its complications, and 4) use of health services.
**Configuration of action-participation groups**	
Study carried out in a rural community in Nigeria, where the maternity service had been out for over on decade and maternal assistance was provided exclusively by traditional attendants (29).	Group of participatory action research group developed a knowledge strategy in two phases: 1) identified maternal mortality as a community problem, analyzed community attitudes, examined factors that contributed to this outcome and possible prevention measures, and 2) planed actions.
**Participatory action cycles**	
Study carried out in 24 neighborhoods in marginal and vulnerable conditions located in India (27).	Groups of women were guided by a facilitator with the following purposes: share personal experiences about maternal health, analyze problems and achievements, increase their knowledge in maternal health, design and implement solutions, and evaluate the success of the actions taken.
**Quality circles**	
Study carried out in Sierra Leone where the access to good quality maternal health services was limited (30).	Community health officers, registered nurses, maternal health attendants, vaccinators, and midwives participated in sequential discussions: 1) cross-learning circles with health professionals and other with midwives to identify problems, 2) cross-learning circles with health-care providers and midwives’ groups to discuss the problems identified in the first discussion, and 3) circles formed to engage district regulatory entities to solve problems identified.
**Emancipation processes**	
Study carried out in a community health center in Canada, located in a vulnerable area (32).	Triads of participants made up by nurses, and mothers and pregnant women from the community were focused on providing community assistance to support pregnancy experiences, reinforce the potential of pregnant women and encourage self-care practices.

### Methodological Quality of Included Studies

The RoB 2.0 cluster tool was used to assess the quality of the randomized controlled trials included in the review. For the outcome of maternal mortality, one study (36) was assessed with high risk due to issues in the *randomization process*. As a result, overall, we assessed this outcome as high risk of bias. In neonatal mortality outcome, two studies (28, 35) were also assessed as risk of bias due to the *timing of identification or recruitment of participants* domain. Overall, this outcome was assessed as high risk of bias. One study (28) was reported as high risk of bias for the perinatal outcome due to the *timing of identification or recruitment of participants* domain. This outcome was judged as high risk of bias. A summary of the judgments is provided in the Supplementary Material S4. No data regarding the outcomes included in our review was provided for the non-randomized. Therefore, we did not apply ROBINS-I to these studies.

Regarding the qualitative studies, we found two studies in which the relationship between the researcher and participants was not clear (30, 40); we found that in one study in which ethical considerations were not expressly manifested (27); and finally, another study whose results were not clearly presented (32). Nevertheless, they fulfilled the other tool’s domains (Supplementary Material S5). Assessment of the certainty of the evidence and its findings, based on the development of meta-synthesis and meta-categories, helped in drawing conclusions with a high, moderate, low, or very low level of confidence using the CerQual approach (Supplementary Material S6).

### Effects of Interventions in Quantitative Studies

Table 3 describes the summary of findings for all outcomes. The full evidence profile with more detailed explanations is available in Supplementary Material S7.

**TABLE 3:** GRADE summary of findings (Bogota, Colombia. 2023).

Knowledge translation strategies compared to no knowledge translation strategies for maternal, neonatal, and perinatal health					
Patient or population: maternal, neonatal, and perinatal health					
Setting: Population					
Intervention: Knowledge translation strategies					
Comparison: No knowledge translation strategies					

Outcomes	No of participants (studies) Follow-up	Certainty of the evidence (GRADE)	Relative effect (95% CI)	Anticipated absolute effects	
				Risk with no knowledge translation strategies	Risk difference with knowledge translation strategies
Maternal mortality	47,510 (4 RCTs) (28, 34, 36, 37)	⊕⊕⊕○ Moderate^a^	RR 0.65 (0.48–0.87)	5 per 1,000	2 fewer per 1,000 (3 fewer to 1 fewer)
Maternal mortality	326 (1 observational study) (33)	⊕⊕⊕○ Moderate^b^ ^,^ ^c^	The study evaluated the impact of a program (Chama’s program) on reducing high maternal mortality rates in Kenya. Compared to controls (n = 115), women in Chamas (n = 211) experienced a lower proportion of maternal deaths (0.9% vs. 1.7%).		
Neonatal mortality	61,231 (5 RCTs) (28, 34–37)	⊕⊕⊕○ Moderate^d^	RR 0.79 (0.70–0.90)	40 per 1,000	8 fewer per 1,000 (12 fewer to 4 fewer)
Neonatal mortality	326 (1 observational study) (33)	⊕⊕⊕○ Moderate^b^ ^,^ ^c^	The study evaluated the impact of a program (Chama’s program) on reducing stillbirth and infant mortality rates in Kenya. Compared to controls, women in Chamas experienced a lower proportion of stillbirths (0.9% vs. 5.2%).		
Perinatal mortality	41,238 (3 RCTs) (28, 36, 37)	⊕⊕⊕○ Moderate^e^	RR 0.84 (0.77–0.91)	64 per 1,000	10 fewer per 1,000 (15 fewer to 6 fewer)
Comunity impact	725 (1 observational study) (39)	⊕⊕⊕○ Moderate^f^	A total of 725 women were interviewed at baseline (intervention *n* = 444; comparison *n* = 281) and 737 at end-line (intervention *n* = 442; comparison *n* = 295). the percentage of women in the intervention area who were aware of at least three danger signs during pregnancy increased from 26% at baseline to 83% at endline, while there was no difference in the comparison area (30% at baseline vs. 28% at endline). Proportion of women in the intervention area who were aware of at least three risks associated with childbirth increased from 24% at baseline to 68% at endline. Comparative area’s knowledge level did not increase. In the intervention area, knowledge of at least three postpartum danger indicators increased by double, while it dropped in the comparison area. Knowing at least three newborn-related risk signs increased from 63% to 83%, with a declining trend in the comparable region area. Additionally, there was a significant increase in the number of danger signs that husbands were aware of, including at least three during pregnancy (6% at baseline vs. 57% at endline), childbirth (11% at baseline vs. 44% at endline), and after childbirth (27% at baseline vs. 77% at endline.		

^a^ High risk of bias due to issues with the randomization process in three out of 4 four studies. All four studies were not blinded regarding individual participants and professionals within clusters.

^b^ High risk of bias due to selection of participants into the study and bias to missing data. The study included participants retrospectively in the control arm preceding the intervention, in the active group. Researchers unintentionally left out the most marginalized women in the community who face difficulties accessing care by including participants from antenatal care facilities. Researchers reported high lost-to-follow-up rates, especially in the control cohort, without explaining this event.

^c^ The study lacked the raw data necessary to evaluate imprecision. The sample included 326 pregnant women in total.

^d^ High risk of bias due to issues with timing of identification or recruitment of participants in relation to timing of randomization two out of 5 studies.

^e^ High risk of bias due to issues with timing of identification or recruitment of participants in relation to timing of randomization in 1 out of 3 studies.

^f^ High risk of bias due to confounding. One study reported a lack of randomization of the intervention clusters.

^g^ The risk in the intervention group (and its 95% confidence interval) is based on the assumed risk in the comparison group and the relative effect of the intervention (and its 95% CI).

GRADE Working Group grades of evidence

High certainty: we are very confident that the true effect lies close to that of the estimate of the effect.

Moderate certainty: we are moderately confident in the effect estimate: the true effect is likely to be close to the estimate of the effect, but there is a possibility that it is substantially different.

Low certainty: our confidence in the effect estimate is limited: the true effect may be substantially different from the estimate of the effect.

Very low certainty: we have very little confidence in the effect estimate: the true effect is likely to be substantially different from the estimate of effect.

CI, confidence interval; RR, risk ratio.

#### Maternal Mortality

**FIGURE 2:**
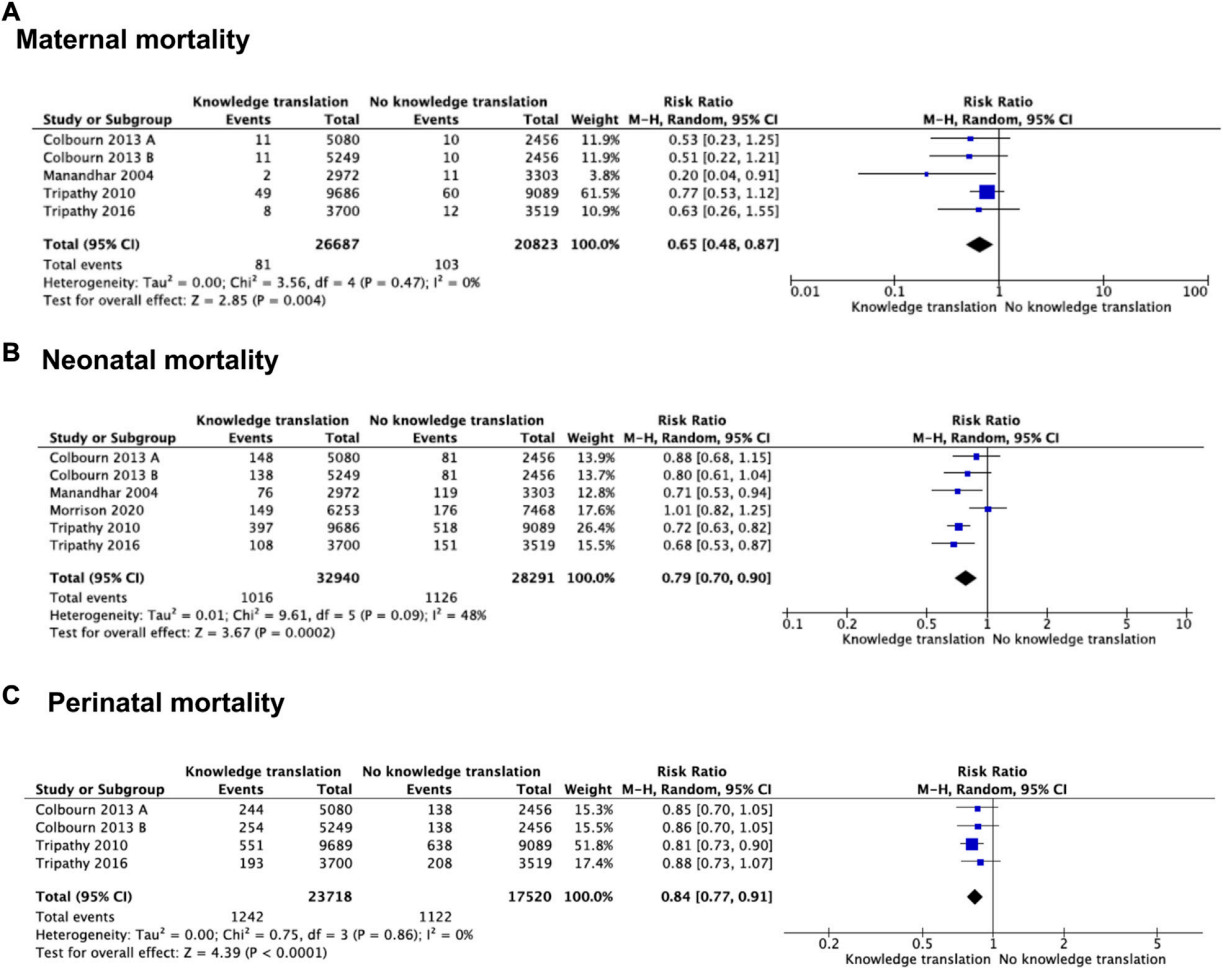
Forest plot of comparison: knowledge translation strategies vs. no knowledge translation strategies, by outcome: **(A)** Maternal mortality, **(B)** Neonatal mortality, **(C)** perinatal mortality (Bogota, Colombia. 2023).

Four studies (*n* = 47,510) compared KTS versus local interventions or no intervention control (27, 35–37). KTS probably reduces maternal mortality compared to control (RR 0.65 95% CI 0.48–0.87; moderate certainty; Figure 2A)). We found no heterogeneity for this outcome (Chi^2^ = 3.56, df = 4, *p* = 0.47; I^2^ = 0%). Sensitivity analysis showed no changes in the effects estimated following the random effects model compared to the fixed effects model (RR = 0.64 95% CI = 0.48–0.86). The certainty of the evidence was assessed moderate due to risk of bias, arising from the randomization process.

One cohort study evaluated a program’s impact (Chama’s program) on reducing high maternal and infant mortality rates in rural western Kenya (33). The program included pregnant women attending their first antenatal control who were or were not exposed to Chama’s program. women in Chamas experienced a lower proportion of maternal deaths (0.9% vs. 1.7%) compared to controls. No additional data was reported. The certainty of the evidence was assessed moderate owing to risk of bias, due to selection of participants and bias due to missing data.

#### Neonatal Mortality

Five studies (*n* = 61,231) evaluated the effects of KTS on neonatal mortality (28, 34–37). Using these KTS probably reduces neonatal mortality compared with no KTS (RR = 0.79 95% CI = 0.70–0.90; moderate certainty; Figure 2B)). We found heterogeneity in this outcome (Chi^2^ = 9.61, df = 5, *p* = 0.09; I^2^ = 48%). We explore the heterogeneity based on the outcome measurement. In two out of five studies, we used as denominator number of deliveries (RR = 0.86 95% CI = 0.61–1.21) (34, 35). We used the number of births for the other three studies (RR = 0.74, 95% CI 0.67–0.82) (28, 36, 37). Subgroup analysis showed an effect modifier for the measurement outcome (test for subgroup differences: Chi^2^ = 0.62, df = 1, *p* = 0.43; I^2^ = 0%). When comparing a fixed effects model with a random effect model, we found that there were no differences with the random effect model. The certainty of the evidence was rated moderate owing to risk of bias, arising from the timing of identification and recruitment of individual participants in relation to timing of randomization.

#### Perinatal Mortality

Three studies (*n* = 41,238) addressed perinatal mortality (28, 36, 37). The effect of KTS compared with no KTS likely reduces perinatal mortality events (RR = 0.84 95% CI = 0.77–0.91; moderate certainty; Figure 2C)). In sensitivity analysis, the pooled estimate was the same for fixed effects compared to random effects. Owing to risk of bias, the certainty of the evidence was rated moderate, arising from the timing of identification and recruitment of individual participants in relation to timing of randomization.

In a cohort study, women that were exposed to an educational program compared with the control group, experienced a lower proportion of stillbirths (0.9% vs. 5.2%) (33). No additional data was reported. The certainty of the evidence was assessed moderate due to risk of bias, arising from selection of participants into the study and bias to missing data.

#### Community Impact

An intervention package was assessed in a quasi-experimental study (39) in women with a history of childbirth in the 12-month period preceding the date of a survey, located in a Bangladesh rural community. The survey was applied to 725 women with a recent history of childbirth at baseline (intervention *n* = 444; comparison *n* = 281), and 737 at endline (intervention *n* = 442; comparison *n* = 295) community. The package included a program to build the capacities of pregnant women through birth preparedness and complication readiness, and the involvement of men specifically, in contributing to a supportive environment for maternal and newborn health. The control group did not receive the package intervention. The program increased the knowledge level of pregnant women and their partners about warning signs during pregnancy (83% intervened group vs. 28% control group), maternal health rights (98% intervened group vs. 40% control group), birth preparedness and complication readiness (78% intervened group vs. 59% control group), and the utilization of prenatal health services (87.1% intervened group vs. 41.5% control group). No additional data was reported. The certainty of the evidence was assessed moderate due to risk of bias, owing to bias due to confounding.

### Effects of Interventions in Qualitative Studies

**FIGURE 3:**
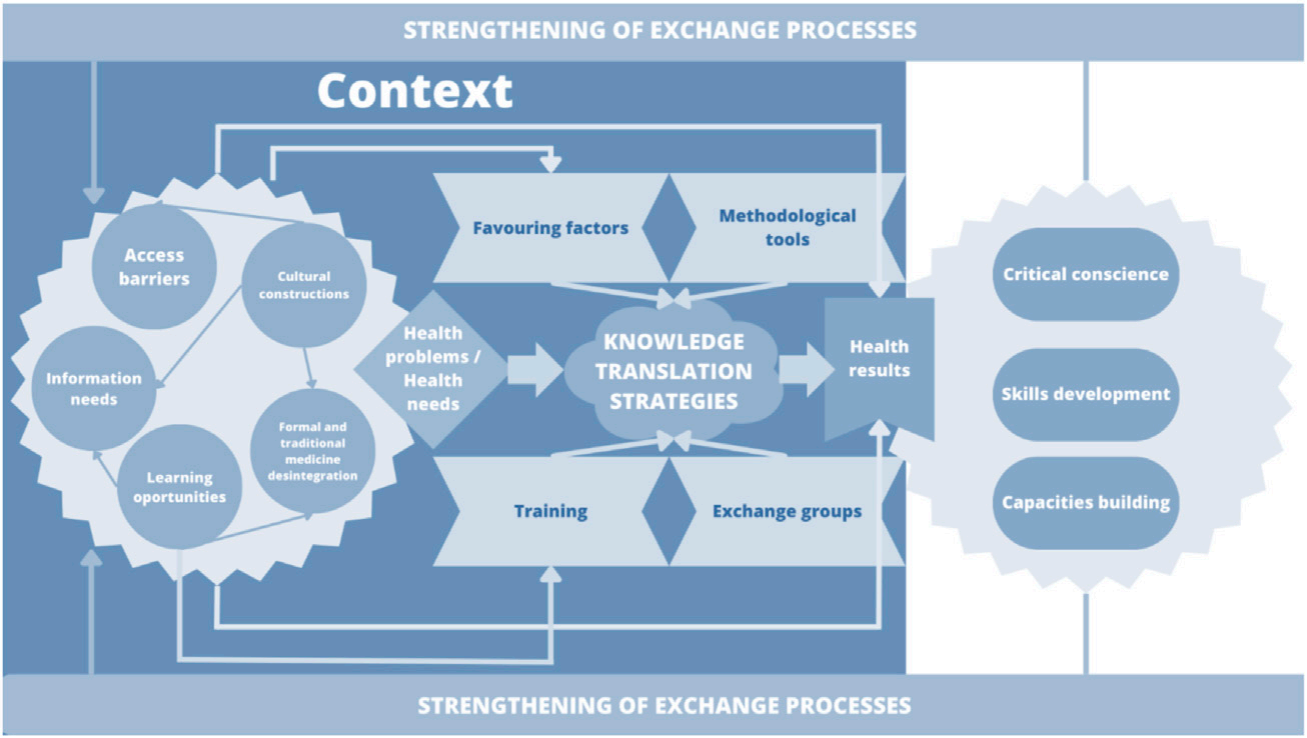
Relationship between findings and synthesis meta-categories (Bogota, Colombia. 2023).

Seven qualitative studies reported data we used to develop the meta-synthesis process (27, 29, 30, 32, 33, 38, 40). From the micro-analysis of the studies data, we obtained 420 substantive codes, which were subsequently analyzed and interpreted based on interpretative codification. Thus, we generated seventeen interpretative synthesis codes (hereinafter called “findings”). Following axial and selective codification steps, we identified three synthesis meta-categories that, from a process logic perspective, represented knowledge translation interventions that produced changes in health (Figure 3).

Under the meta-category one, context elements for KTS, we grouped the elements that were part of community and knowledge translation processes for improving maternal and perinatal health. These elements were not sequential, but they can be reciprocally related. Context was determinant for maternal health outcomes; therefore, this meta-category includes the conditions of the territory that generate access barriers, the central elements of health interventions and their importance for the empowerment of communities in relation to territorial transformation processes. Inclusion and optimization of the access to better living conditions, based on basic sanitization and educational infrastructure, were the key contexts for defining the barriers to access maternal and perinatal knowledge (32, 40). The value attributed to the knowledge of midwives, which are consider as not derived from scientific knowledge, and the meaning of their importance and availability in scattered territories constituted a significant factor for integrating their work into the health system (30, 40). There is no doubt that such value helps in the identification of translation and learning opportunities in areas that are regarded as increasingly relevant for generating better care practices for women (30, 32).

In the meta-category two, experiences translation strategies, the KTS contributed with the improvement of maternal, neonatal, and perinatal outcomes at multiple healthcare levels among professionals, traditional birth practitioners and the community. The idea was to transform the limited knowledge on various health issues. These strategies were characterized by the empirical knowledge of people from the community, such as community healthcare workers, traditional midwives, women of childbearing age, older women, and community leaders (27, 29–32). These studies emphasized that techniques such as reinterpretation of knowledge about the causes of maternal mortality together with reinforcement of non-detrimental cultural practices were particularly useful for optimizing healthcare (30, 38, 40). In addition, the use of hypothetical scenarios based on questions as ‘what is the risk of home birth?’, and knowledge supporting picture cards were well accepted within the dialog processes between several population studies (29, 39). Facilitators in these knowledge translation strategies were mainly local women (29, 39). Communicated in dialog with communities, promote the confidence and process sustainability, respect for local practices and an open interaction focused on listening, avoiding negative criticism, increased acceptability and fostered learning (30, 38, 40). Among groups, the experience of women who had children was crucial in these interactions. The recurrent communication of the program’s advances and individual responsibilities was successfully implemented to maintain the interest of participants (31).

The meta-category three, health results, demonstrated the capacity generated by community working groups to solicit assistance to improve community health and social structures with the objective of preventing maternal mortality (39). The group meetings proved to their participants that their involvement in collective problem solving develops a sense of togetherness and increases their chances of bringing about changes in healthcare (32, 38, 40). Critical awareness was raised in communities following the strengthening of learning competences and resource mobilization to develop skills for situation analysis and analysis of political opportunities (31).

As healthcare providers developed a relationship with midwives or facilitators, they ensured a climate of cooperation among members of the healthcare system (32). This was achieved by increasing their credibility and community support, improving women’s confidence, autonomy and empowering them in their role as mediators with access to information (30). In summary, the relationship between midwives and healthcare providers was changed after strengthening organizational skills such as problem-solving.

## Discussion

This systematic review, meta-analysis and meta-synthesis included fourteen studies that describe eight types of KTS aiming to improve maternal, neonatal, and perinatal outcomes at professional, technical, and traditional levels. Our findings suggest that in groups exposed to KTS, compared to control groups, there is a reduction in maternal mortality, neonatal mortality, and perinatal mortality with moderate certainty of the evidence. Our results also showed increased knowledge of pregnant women and their partners about warning signs during pregnancy, maternal health rights, birth preparedness, and complication readiness, and improved healthcare services utilization. We did not find evidence that describes the effect of KTS on other maternal, neonatal, and perinatal outcomes previously defined in our protocol.

Qualitative evidence-informed strategies that supported knowledge translation strategies among professionals, non-professionals, and communities, recognizing the need to have a bidirectional communication of experiences, perceptions, and practices between them. The qualitative studies included in this SR provided evidence that identified the lack of knowledge to identify causes and complications during pregnancy, childbirth, and perinatal periods in women and midwives and in the communities to which they belong. Furthermore, our findings show that the gaps in knowledge and practices produce maternal deaths. Consequently, it is necessary to jointly articulate maternal and child healthcare between the institutional and community actors considered by our research. It must be based on confidence relationships, and training processes oriented to improvement in listening and communication skills, leadership, management of socio-economic resources, characteristics of labor, identification of safe delivery, delays, risks, cultural perspectives in childbirth care, and general aspects of maternal and perinatal care. It will contribute to the strengthening of learning and generate confidence and autonomy in decision-making.

Strategies derived from the quantitative studies, were frequently consistent with most of our findings from the qualitative meta-synthesis. Although it is recognized that these findings pertain to specific community contexts, these results can shed light on some elements that policymakers and stakeholders should consider implementing program policies and interventions at the community level.

In 2007, Haws et al. (42) searched systematically for studies that evaluated impact of a three interventions, family-community, outreach, or facility-based clinical care during the antenatal, intrapartum, and postnatal periods. The systematic review included 41 studies, and they did not identify studies at a health systems level that measure the effectiveness of intervention packages. 14 out of 41 studies integrated an intervention that linked communities with healthcare systems, and one study (34) was included in our review. However, most of the studies included in Haws’ et al review incorporated interventions that involved vertical community engagement like traditional birth attendant training programs or trained healthcare workers. Additionally, those primary studies that took into consideration horizontal engagement did not include a comparison group. We focused on studies that involved community bidirectional interactions between organizations and community members.

In 2010, Schiffman et al. (43) carried out a systematic review for community-based intervention packages (CBIPs) in rural settings for improving perinatal health in developing countries. The systematic review focused on specific community strategies such as family-community care, outreach services, and facility-based clinical care, included nine studies, 6 RCTs and three non-randomized studies. Only one study was included in our review (34). Although Schiffman et al did not conduct a metanalysis, like our systematic review, they reported a benefit of CBIPs compared to control group, on neonatal and perinatal mortality. Schiffman´s systematic review included different studies than ours because they included community-based interventions that did not involve horizontal engagement between local community and organizations.

Other four systematic reviews that conducted metanalysis (44–47) reported findings of different knowledge translation strategies in reducing maternal and neonatal morbidity and mortality. Specific interventions included in these systematic reviews were home visit for neonatal care (44); traditional birth attendant (TBA) training (47); women’s groups practicing participatory learning and action (46); and CBIPs (45). Two systematic reviews (44, 46) included only randomized controlled trials, and the other two studies (45, 47), in addition to RCTs, included quasi-randomized controlled trials. Compared to our findings, we also found a significant effect of KTS on maternal, neonatal, and perinatal mortality.

However, strategies included on these four systematic reviews included vertical and horizontal communication between communities and healthcare services. Our interest was only focused on bidirectional communication processes.

### Strengths and Limitations

This systematic review has several strengths. We established a robust methodological process with explicit eligibility criteria and assessed eligibility. Data were extracted in duplicate. Further, we included in our systematic search five online databases, five online platforms and library catalogues. We rated the certainty of the body of evidence applying guidance from the GRADE Working Group. Moreover, we included both quantitative and qualitative studies qualitative findings gave a deep interpretation of KTS identified by quantitative studies. We compared KTS with a control group to broaden the findings and applicability of our results in maternal, neonatal, and perinatal population. We also identified an additional RCT that were not reported in other systematic reviews.

Our systematic review has some limitations, mostly inherent in the evidence. The significant number of studies whose full text was not retrieved generates uncertainty about the total number of KTS included in this review. However, we accessed several national electronic databases and contacted the corresponding authors without obtaining a response. No outcomes were included in the evidence that evaluated the possible disadvantages caused by KTS, which leads to uncertainty in the balance of benefits and harms that these strategies may produce at community level. The studies we included in our review described various KT methodologies, which may have led to clinical heterogeneity in our findings. Since these KTS will be used in a community, researchers must consider how broadly applicable our findings are. Furthermore, the collected evidence does not show knowledge translation strategies implemented in urban or semi-urban contexts; or other interventions based on mHealth. This aspect is relevant to the change in health services after the pandemic scenario. Finally, the assessment of risk of bias in these studies suggests concerns regarding serious considerations about the susceptibility to bias in most of the studies.

### Implications of the Results for Practice, Policy, and Future Research

Our qualitative findings suggest that interventions based on knowledge translation must consider the context. So, precarious structural and intermediate health determinants do not allow transformations in women’s healthcare and their child’s. Additionally, Community Health Worker’s recognition can improve quality and opportunity in attention in rural or remote health systems. Public health researchers, practitioners, and policymakers should consider the resource’s availability due to the influence of the sustainability of these interventions.

Knowledge translation processes at different levels can improve maternal, neonatal, and perinatal health in rural communities. Future research must include other patient-important outcomes. We were able to observe that these processes were not documented in the literature; therefore, more research will be required to determine the impact of KTS on the maternal, neonatal, and perinatal outcomes that were proposed during our review and could not be assessed through empirical evidence. Other significant research must consider the difference between urban and rural areas, or the affordability of interventions based on mHealth.

### Conclusion

This systematic review, meta-analysis and meta-synthesis suggests evidence on the potential benefits of KTS in reducing maternal, neonatal, and perinatal mortality. It also points out the essential elements of knowledge strategies that can be incorporated and adapted in different circumstances where exist horizontal community and heath care services are engaged. To replicate the strategies identified in this review, it is necessary to consider the context and specific characteristics of the territories and communities.
