# Supplementary material for: Community-Based Knowledge Translation Strategies for Maternal, Neonatal, and Perinatal Outcomes: A Systematic Review of Quantitative and Qualitative Data
Source: Int J Public Health. 2023 Apr 20;68:1605239. doi: 10.3389/ijph.2023.1605239 (PMC10157638; doi:10.3389/ijph.2023.1605239)

**Supplementary material 4. Risk of bias judgments per outcomes in randomized controlled trials and non-randomized studies**

***Randomized controlled trial***

**Maternal mortality**


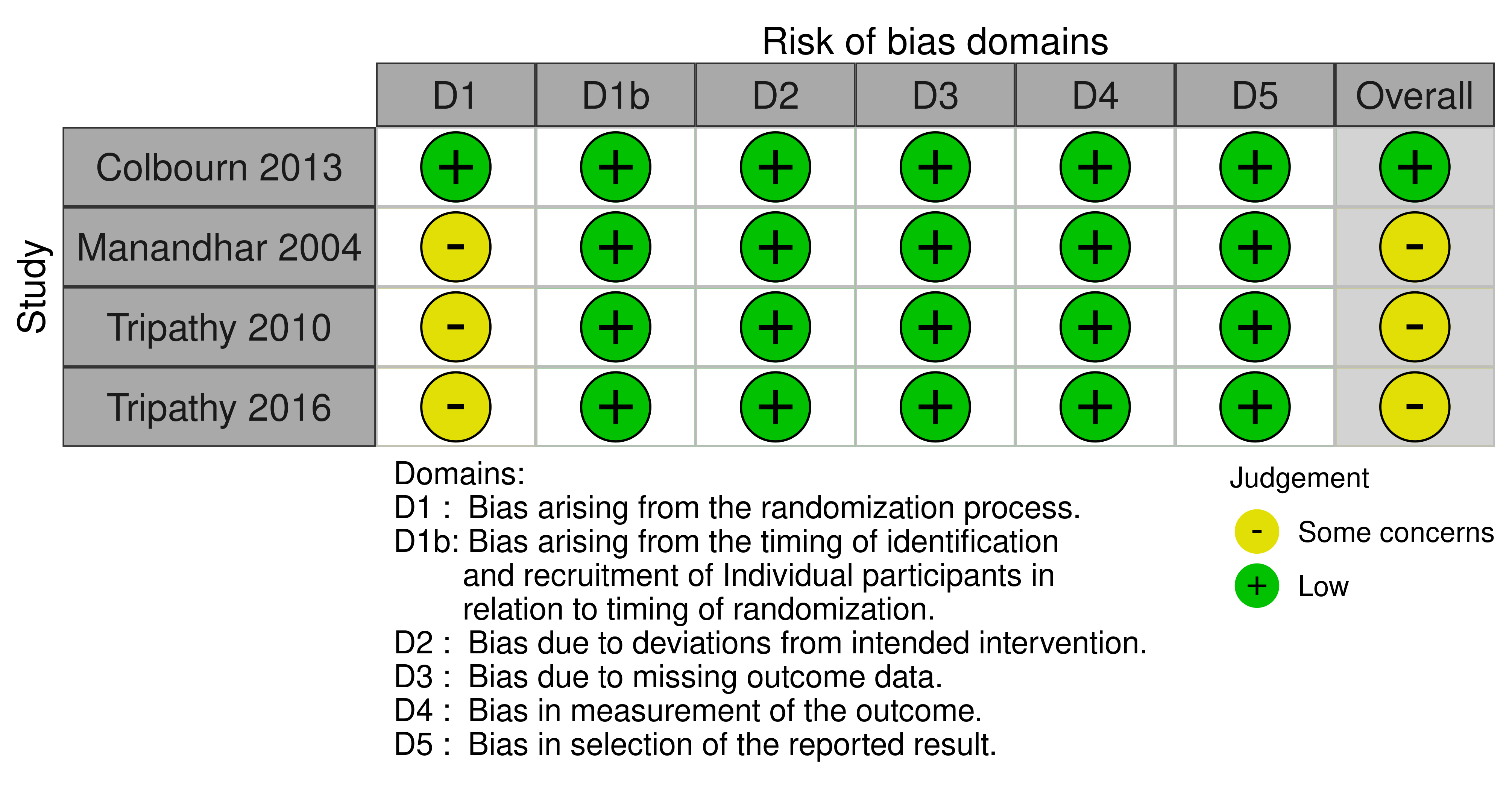


**Neonatal mortality**

**
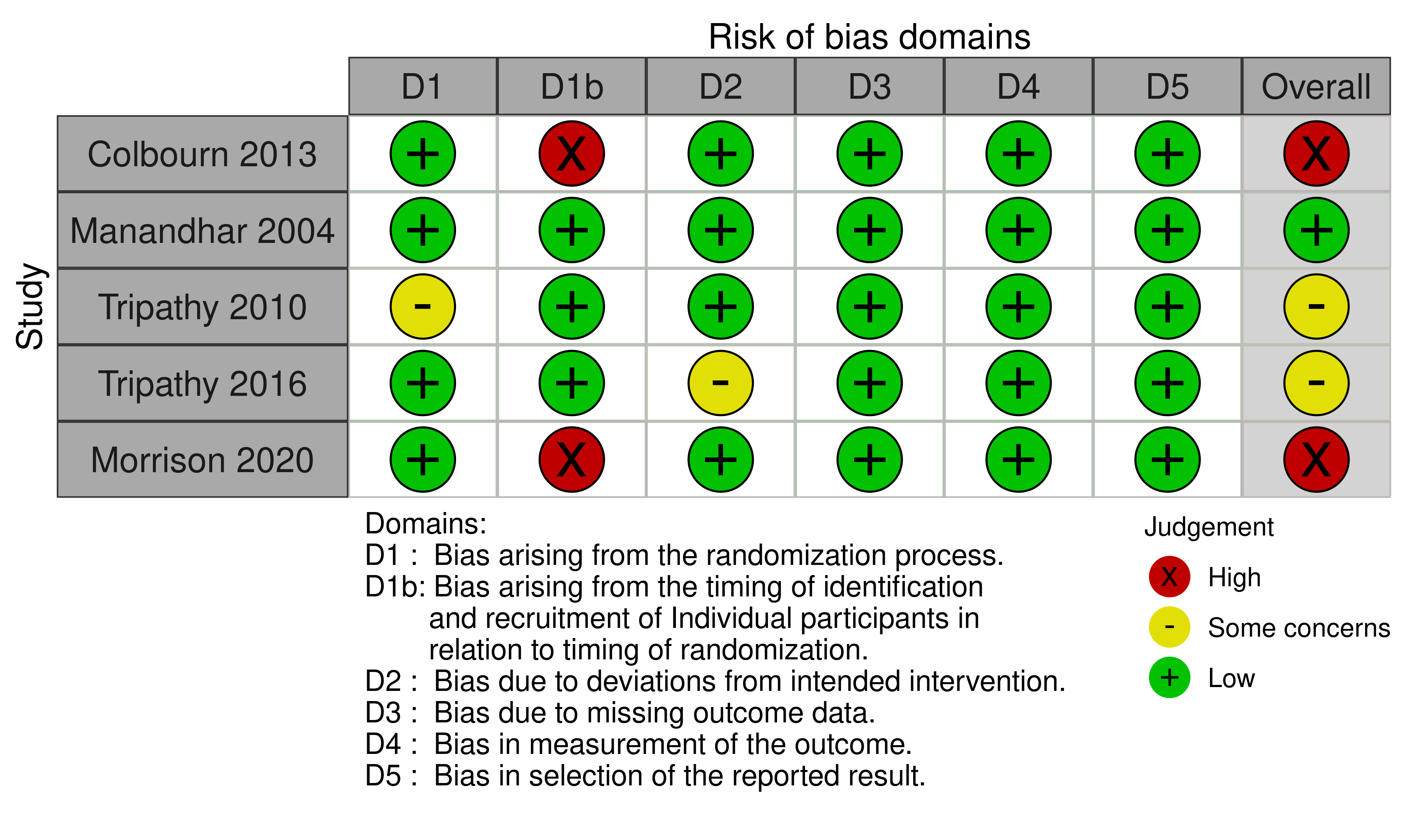
**

**Perinatal mortality**

**
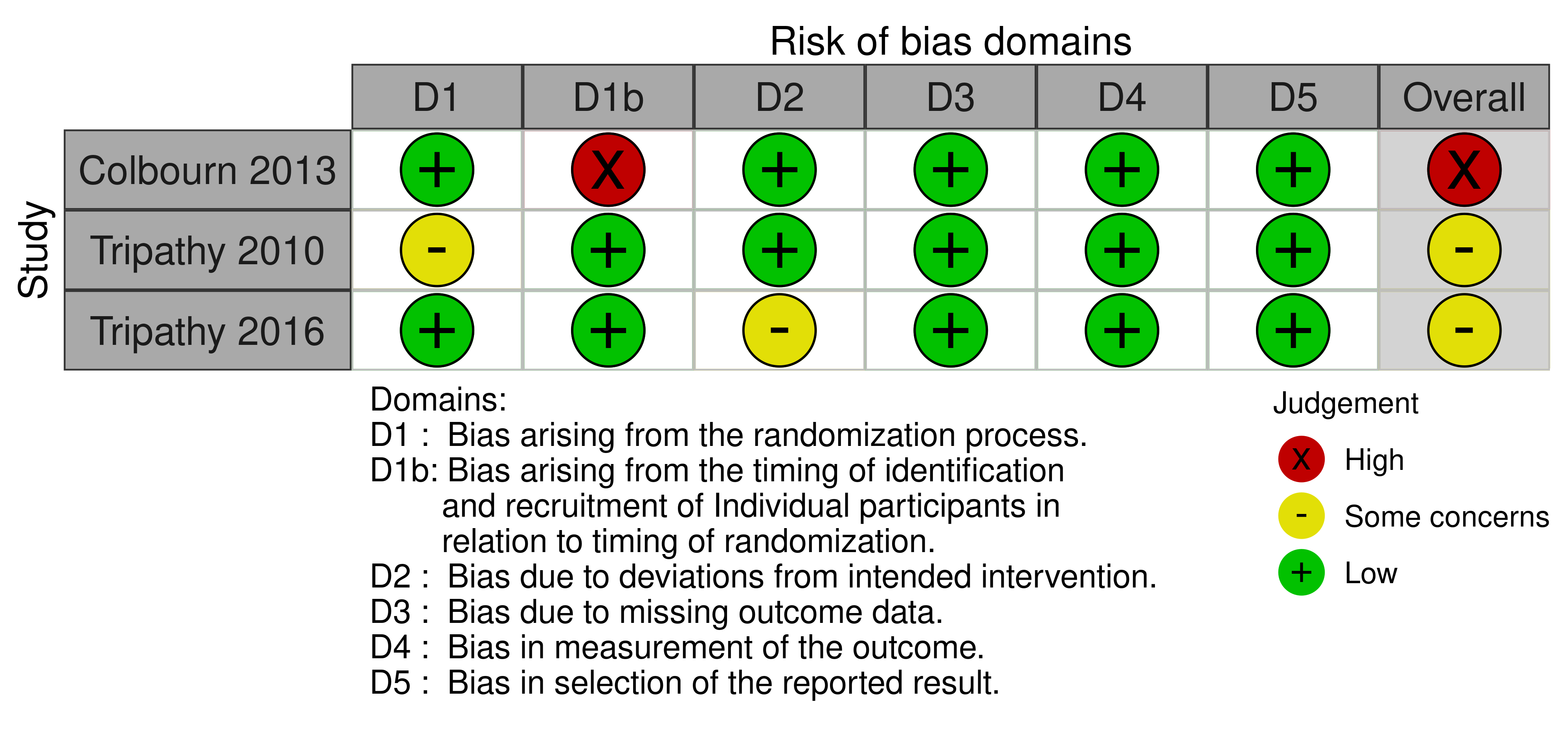
**

***Non-randomized controlled studies***

**Maternal mortality**


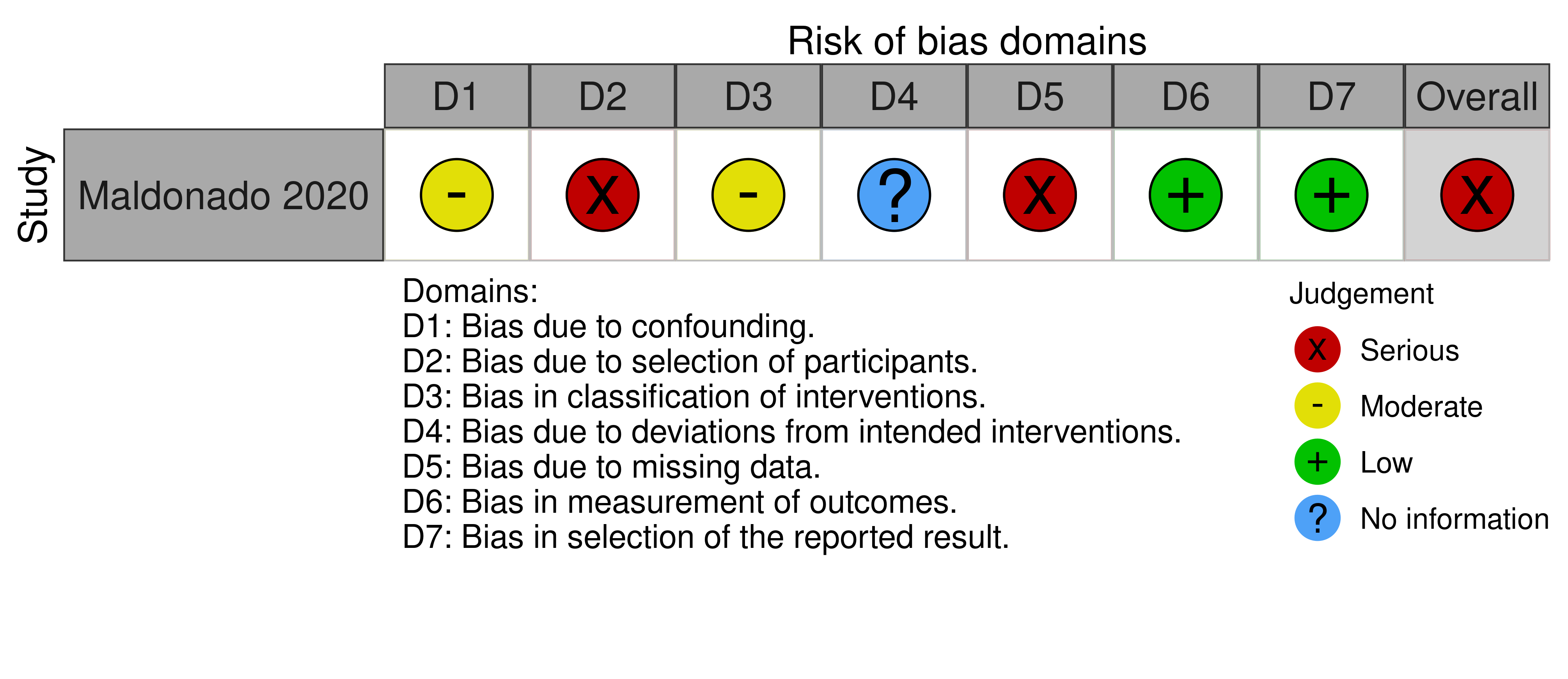


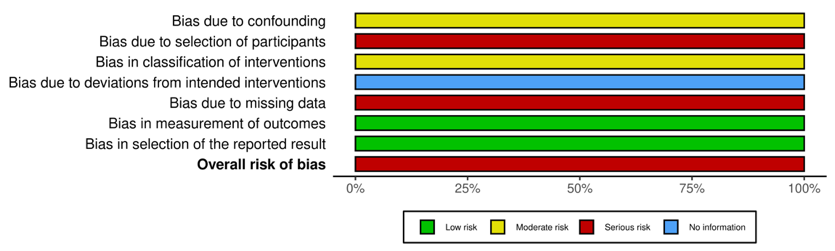


**Perinatal mortality**


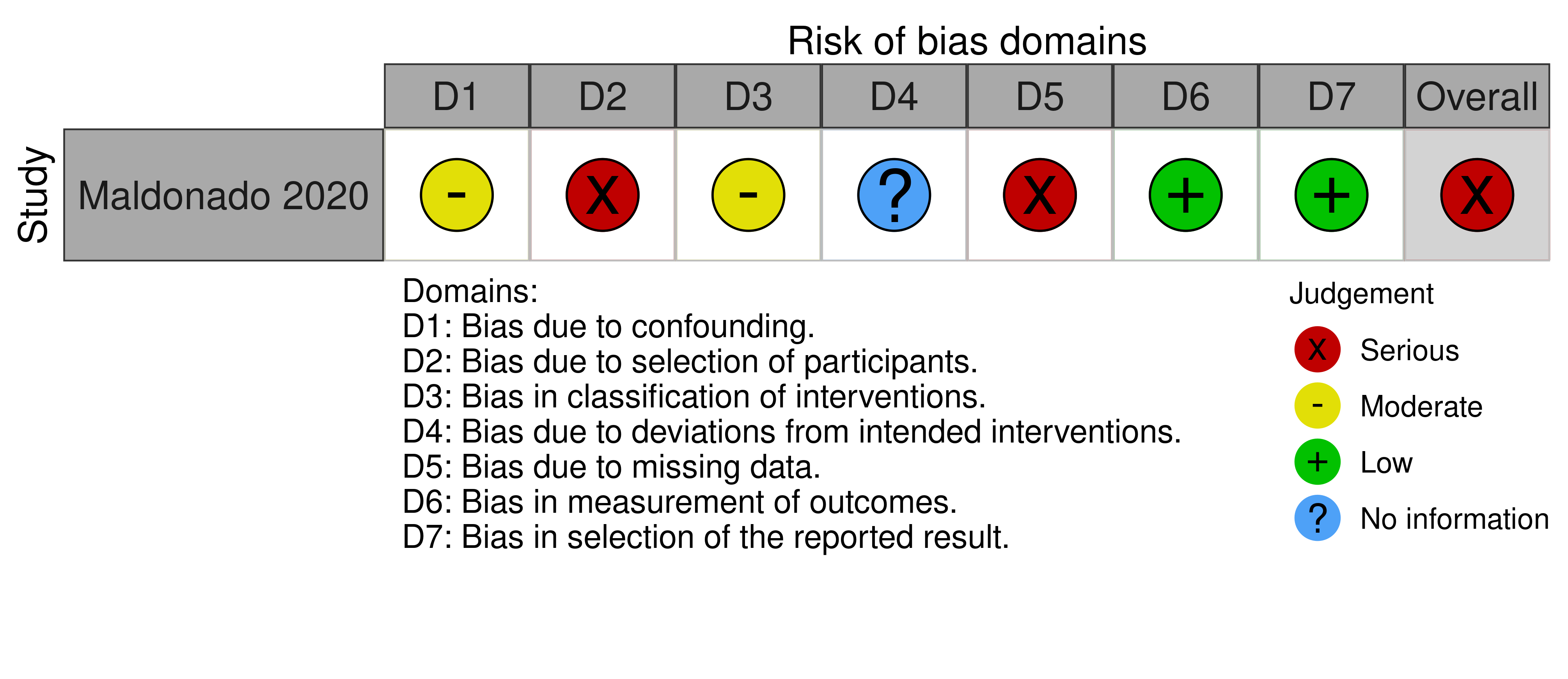


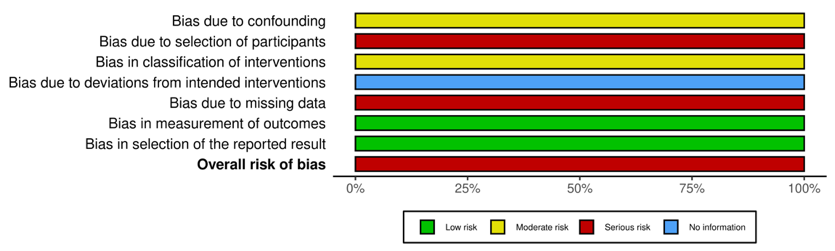


**Community impact**


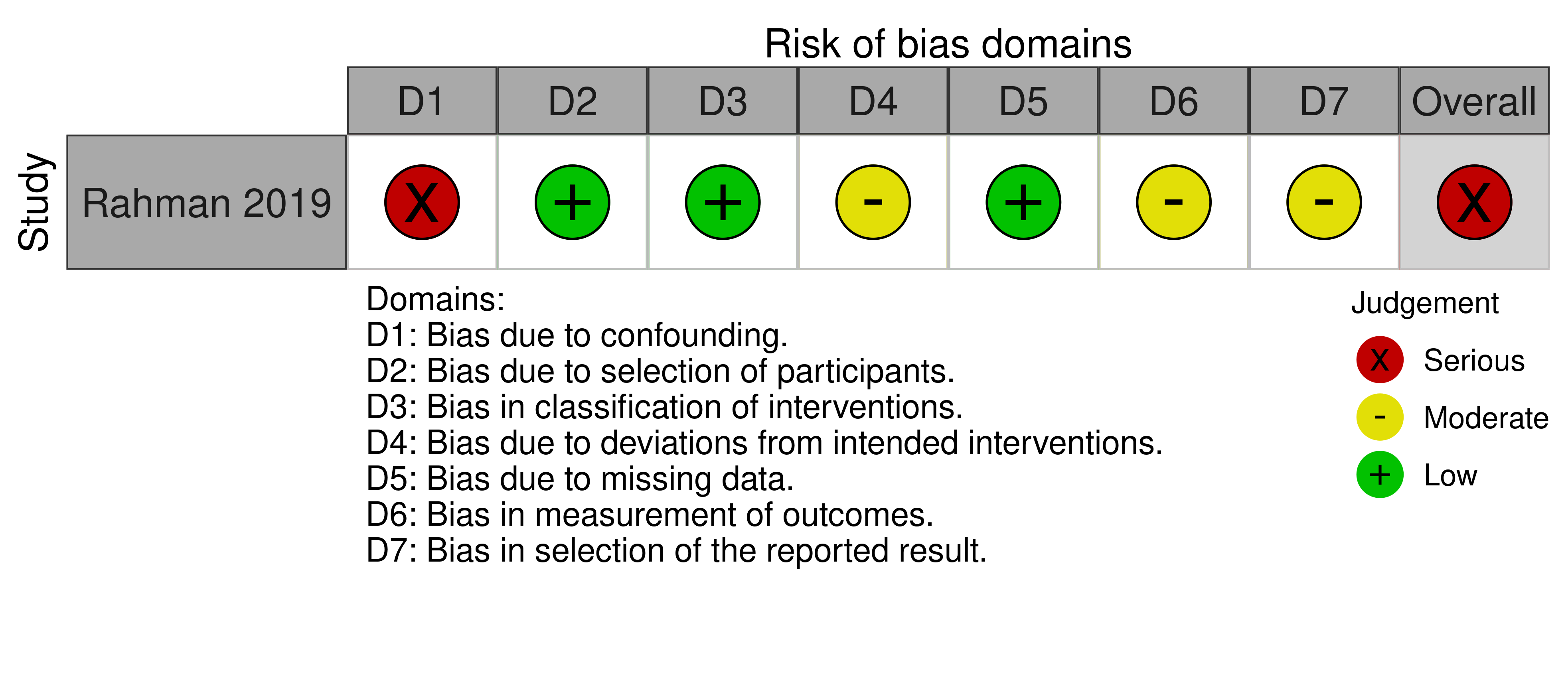


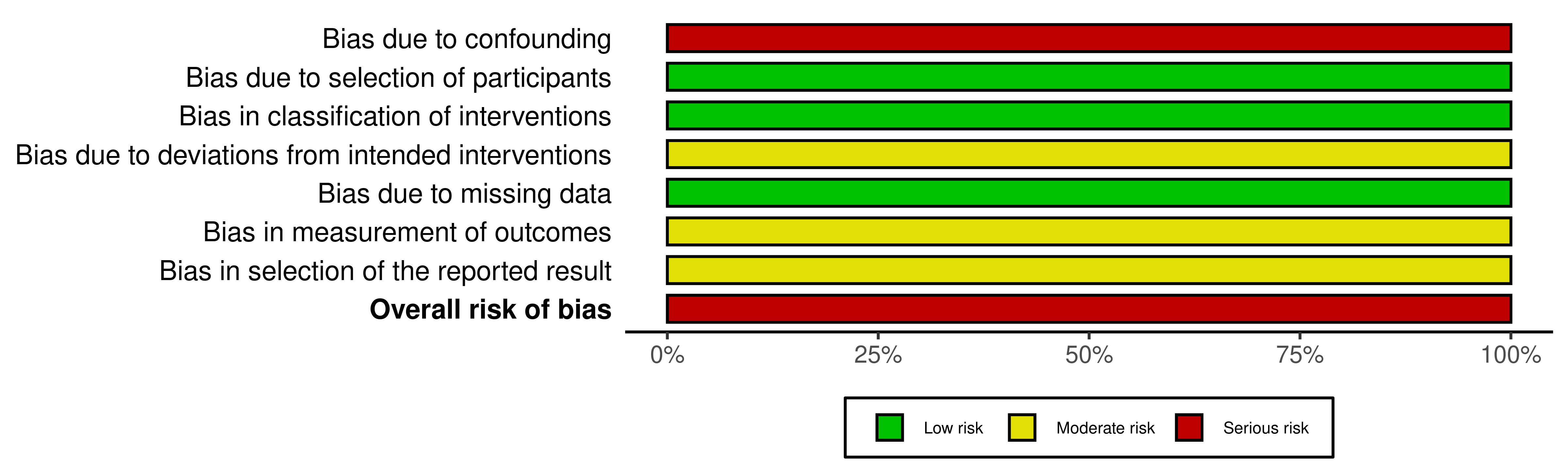

Supplement: Supplementary file 1 [file DataSheet4.docx]
