# Supplementary material for: Community-Based Knowledge Translation Strategies for Maternal, Neonatal, and Perinatal Outcomes: A Systematic Review of Quantitative and Qualitative Data
Source: Int J Public Health. 2023 Apr 20;68:1605239. doi: 10.3389/ijph.2023.1605239 (PMC10157638; doi:10.3389/ijph.2023.1605239)
Supplement: Supplementary file 2 [file DataSheet7.docx]

**Supplementary material 7. Evidence profile table**

| **Certainty assessment** | | | | | | | **№ of patients** | | **Effect** | | **Certainty** | **Importance** |
| --- | --- | --- | --- | --- | --- | --- | --- | --- | --- | --- | --- | --- |
| **№ of studies** | **Study design** | **Risk of bias** | **Inconsistency** | **Indirectness** | **Imprecision** | **Other considerations** | **knowledge translation strategies** | **no knowledge translation strategies** | **Relative** **(95% CI)** | **Absolute** **(95% CI)** |  |  |
| **Maternal mortality** | | | | | | | | | | | | |
| 4^1,2,3,4^ | randomised trials | serious^a^ | not serious | not serious | not serious | none | 81/26687 (0.3%) | 103/20823 (0.5%) | **RR 0.65** **(0.48 to 0.87)** | **2 fewer per 1,000** **(from 3 fewer to 1 fewer)** | ⨁⨁⨁◯ Moderate | CRITICAL |
| **Maternal mortality** | | | | | | | | | | | | |
| 1^5^ | observational studies | serious^b^ | not serious | not serious | not serious^c^ | none | The study evaluated the impact of a program (Chama's program) on reducing high maternal mortality rates in Kenya. Compared to controls (n=115), women in Chamas (n= 211) experienced a lower proportion of maternal deaths (0.9% vs. 1.7%). | | | | ⨁⨁⨁◯ Moderate | CRITICAL |
| **Neonatal mortality** | | | | | | | | | | | | |
| 5^1,2,3,4,6^ | randomised trials | serious^d^ | not serious | not serious | not serious | none | 1016/32940 (3.1%) | 1126/28291 (4.0%) | **RR 0.79** **(0.70 to 0.90)** | **8 fewer per 1,000** **(from 12 fewer to 4 fewer)** | ⨁⨁⨁◯ Moderate | CRITICAL |
| **Neonatal mortality** | | | | | | | | | | | | |
| 1^5^ | observational studies | serious^b^ | not serious | not serious | not serious^c^ | none | The study evaluated the impact of a program (Chama's program) on reducing stillbirth and infant mortality rates in Kenya. Compared to controls, women in Chamas experienced a lower proportion of stillbirths (0.9% vs. 5.2%). | | | | ⨁⨁⨁◯ Moderate | CRITICAL |
| **Perinatal mortality** | | | | | | | | | | | | |
| 3^1,3,4^ | randomised trials | serious^e^ | not serious | not serious | not serious | none | 1242/23718 (5.2%) | 1122/17520 (6.4%) | **RR 0.84** **(0.77 to 0.91)** | **10 fewer per 1,000** **(from 15 fewer to 6 fewer)** | ⨁⨁⨁◯ Moderate | CRITICAL |
| **Community impact** | | | | | | | | | | | | |
| 1^7^ | observational studies | serious^f^ | not serious | not serious | not serious | none | A total of 725 women were interviewed at baseline (intervention n= 444; comparison n= 281) and 737 at end-line (intervention n= 442; comparison n= 295). the percentage of women in the intervention area who were aware of at least three danger signs during pregnancy increased from 26% at baseline to 83% at endline, while there was no difference in the comparison area (30% at baseline vs 28% at endline). Proportion of women in the intervention area who were aware of at least three risks associated with childbirth increased from 24% at baseline to 68% at endline. Comparative area's knowledge level did not increase. In the intervention area, knowledge of at least three postpartum danger indicators increased by double, while it dropped in the comparison area. Knowing at least three newborn-related risk signs increased from 63% to 83%, with a declining trend in the comparable region area. Additionally, there was a significant increase in the number of danger signs that husbands were aware of, including at least three during pregnancy (6% at baseline vs. 57% at endline), childbirth (11% at baseline vs. 44% at endline), and after childbirth (27% at baseline vs. 77% at endline. | | | | ⨁⨁⨁◯ Moderate | CRITICAL |

**CI:** confidence interval; **RR:** risk ratio

#### Explanations

a. High risk of bias due to issues with the randomization process in 3 out of 4 four studies. All four studies were not blinded regarding individual participants and professionals within clusters.

b. High risk of bias due to selection of participants into the study and bias to missing data. The study included participants retrospectively in the control arm preceding the intervention, in the active group. Researchers unintentionally left out the most marginalized women in the community who face difficulties accessing care by including participants from antenatal care facilities. Researchers reported high lost-to-follow-up rates, especially in the control cohort, without explaining this event.

c. The study lacked the raw data necessary to evaluate this domain. The sample included 326 pregnant women in total.

d. High risk of bias due to issues with timing of identification or recruitment of participants in in relation to timing of randomization 2 out of 5 studies.

e. High risk of bias due to issues with timing of identification or recruitment of participants in relation to timing of randomization in 1 out of 3 studies.

f. High risk of bias due to confounding. One study reported a lack of randomization of the intervention clusters.

#### References

1.Colbourn, T. Nambiar B. Bondo A. Makwenda C. Tsetekani E. Makonda-Ridley A. Costello A.. Effects of quality improvement in health facilities and community mobilization through womens groups on maternal, neonatal and perinatal mortality in three districts of Malawi: MaiKhanda, a cluster randomized controlled effectiveness trial. International Health; 2013.

2.Manandhar, D. S. Osrin D. Shrestha B. P. Mesko N. Morrison J. Tumbahangphe K. M. Costello A. M.. Effect of a participatory intervention with womens groups on birth outcomes in Nepal: cluster-randomised controlled trial. The Lancet; 2004.

3.Tripathy, P. Nair N. Sinha R. Rath S. Gope R. K. Rath S. Prost A.. Effect of participatory womens groups facilitated by Accredited Social Health Activists on birth outcomes in rural eastern India: A cluster-randomised controlled trial. The Lancet Global Health; 2016.

4.Tripathy, P. Nair N. Barnett S. Mahapatra R. Borghi J. Rath S. Costello A.. Effect of a participatory intervention with womens groups on birth outcomes and maternal depression in Jharkhand and Orissa, India: A cluster-randomised controlled trial. The Lancet; 2010.

5.Maldonado LY, Songok JJ,Snelgrove JW,Ochieng CB,Chelagat S,Ikemeri JE,Okwanyi MA,Cole DC,Ruhl LJ,Christoffersen-Deb A.. Promoting positive maternal, newborn, and child health behaviors through a group-based health education and microfinance program: a prospective matched cohort study in western Kenya. BMC Pregnancy Childbirth; 2020.

6.Morrison J, Tumbahangphe K Sen A Gram L Budhathoki B Neupane R et al.. Health management committee strengthening and community mobilisation through womens groups to improve trained health worker attendance at birth in rural Nepal: a cluster randomised controlled trial. BMC pregnancy and childbirth; 2020.

7.Rahman, A. E.,Perkins,J.,Mazumder,T.,Haider,M. R.,Siddique,A. B.,Capello,C.,Santarelli,C.,& El Arifeen,S.. Capacities of women and men to improve maternal and newborn health: Effect of a community-based intervention package in rural Bangladesh. Journal of global health; 2019.
