# Supplementary material for: Community-Based Knowledge Translation Strategies for Maternal, Neonatal, and Perinatal Outcomes: A Systematic Review of Quantitative and Qualitative Data
Source: Int J Public Health. 2023 Apr 20;68:1605239. doi: 10.3389/ijph.2023.1605239 (PMC10157638; doi:10.3389/ijph.2023.1605239)
Supplement: Supplementary file 3 [file DataSheet6.docx]

## **Supplementary material 6. Summary of Qualitative Findings Table**

| **#** | **Summarized review finding** | **GRADE-CERQual Assessment of confidence** | **Explanation of GRADE-CERQual Assessment** | **References** |
| --- | --- | --- | --- | --- |
| **CONTEXTUAL ELEMENTS FOR KNOWLEDGE EXCHANGE** | | | | |
| 1 | Problems of integration of formal and traditional knowledge Health problems in the communities were determined by the conditions of the context. (39, 40). Feelings of insecurity, early motherhood, and loneliness stand out (35). The integration strategies of traditional care with health services, as they were not consensual, turned out to be a problem that divided communities and women seeking care. Therefore, it is necessary to jointly articulate these processes (35- 40, 42) | Moderate confidence | Moderate concerns regarding methodological limitations, No/Very minor concerns regarding coherence, No/Very minor concerns regarding adequacy, and Moderate concerns regarding relevance | Lapierre J, et. al. 2006; Alcock GA, et. al. 2009; Esienumoh EE et. al. 2018; Higgins-Steele A, et. al. 2015; Rath S, et. al. 2010; Joseph C, et.al. 2020; Sarmiento I, et.al. 2021 |
| 2 | The experiential knowledge of the midwives (recognized by them as not derived from scientific knowledge), represented an essential element for the integration of their work with the health systems available in the territory (35, 36). For this, barriers such as the lack of knowledge about issues such as the main direct causes of maternal death, hygiene practices for delivery care, and the improvement of relationships between traditional and formal professionals must be overcome (35, 36, 37). To strive for the integration of experiences and knowledge between the actors of the health systems and the midwives (health agents) would result in better health results if one considers that in the most dispersed territories, the health agents are the ones who solve the problems. The health needs of the population to which they belong and are culturally recognized for it (36, 37) | Moderate confidence | Moderate concerns regarding methodological limitations, No/Very minor concerns regarding coherence, No/Very minor concerns regarding adequacy, and Moderate concerns regarding relevance | Alcock GA et al. 2009; Esienumoh EE et al. 2018; Higgins-Steele A et al. 2015; Rath S et al. 2010; Joseph C, et.al. 2020; Sarmiento I, et.al. 2021 |
| 3 | Cultural constructions represented an important element in the population's understanding of maternal perinatal health (35, 36, 38, 42). On the one hand, the construction of representative identities about the people in the community who were part of the action processes in favor of improving the health of women (35, 36), and on the other, the understanding of pregnancy and death as determining elements of pregnancy outcomes. (38, 42) | Moderate confidence | Minor concerns regarding methodological limitations, No/Very minor concerns regarding coherence, No/Very minor concerns regarding adequacy, and Minor concerns regarding relevance | Lapierre et al. 2006; Alcock GA et al. 2009; Esienumoh EE et al. 2018; Rath S et al. 2010; |
| 4 | The existence of a gap between the health system and traditional forms of care is highlighted which generate feelings of fear and make it difficult to access timely care (37). Consequently, follow-up programs are proposed with peripheral actors preferably from the community, so that integration can be better received through processes of acceptance and change (38) | High confidence | Minor concerns regarding methodological limitations, No/Very minor concerns regarding coherence, No/Very minor concerns regarding adequacy, and Minor concerns regarding relevance | Lapierre et al. 2006; Higgins-Steele A et al. 2015; |
| 5 | Factors of the physical environment were recognized as precursors to maternal complications and deaths (36). Its inclusion both in the analysis of results and in the generation and territorial transformation was important in at least three of the selected studies. (36, 38 - 40). | Moderate confidence | Minor concerns regarding methodological limitations, No/Very minor concerns regarding coherence, No/Very minor concerns regarding adequacy, and Minor concerns regarding relevance | Lapierre et al. 2006; Esienumoh EE et al. 2018; Rath S et al. 2010; Joseph C, et.al. 2020; Sarmiento I, et.al. 2021 |
| 6 | The lack of information on the causes of maternal deaths and morbidities was common, both among traditional actors and among women in the communities, this, in turn, contributes to the type of care that midwives provide or women seek (35, 36, 40) | Moderate confidence | Minor concerns regarding methodological limitations, No/Very minor concerns regarding coherence, No/Very minor concerns regarding adequacy, and Minor concerns regarding relevance | Alcock GA et al. 2009; Esienumoh EE et al. 2018; Joseph C, et.al. 2020; Sarmiento I, et.al. 2021 |
| **HEALTH OUTCOMES** | | | | |
| 7 | The training processes strengthen capacities and abilities. Its success stems from community participation since it has a greater impact on the implementation of knowledge translation strategies (36). Among the topics of the training processes, there is an improvement in listening and communication skills, characteristics of labor, identification of safe delivery, delays, risks, cultural perspectives in childbirth care, and general aspects of maternal care, and perinatal (37). The training processes were focused on "specific audiences" and separately: community workers and communities. (36, 37, 39, 40). | Moderate confidence | Minor concerns regarding methodological limitations, No/Very minor concerns regarding coherence, No/Very minor concerns regarding adequacy, and Minor concerns regarding relevance | Esienumoh EE et al. 2018; Higgins-Steele A et al. 2015; Rath S et al. 2010; Joseph C, et.al. 2020; Sarmiento I, et.al. 2021 |
| 8 | The configuration of cooperative groups, together with intersectoral cooperation strategies, bonds of friendship and trust, and the recognition of leadership, are factors that favor knowledge exchange strategies (35, 37). However, these strategies, from the beginning, must have the participation of the communities. (35, 37, 42). | Moderate confidence | Minor concerns regarding methodological limitations, No/Very minor concerns regarding coherence, No/Very minor concerns regarding adequacy, and Minor concerns regarding relevance | Lapierre et al. 2006; Alcock GA et al. 2009; Higgins-Steele A et al. 2015; Rath S et al. 2010; |
| 9 | The discussion and knowledge translation groups are characterized by the voluntary incorporation of people from the communities, with different expertise in planning strategies and the recognition and representation of diverse cultural constructs (35 -38). The discussion and exchange groups guarantee the identification of needs, the development of proposals for change and improvement, the resolution of problems, and the appropriation and substantivity of actions to improve maternal-perinatal and neonatal health. (35-38, 42) | Moderate confidence | Minor concerns regarding methodological limitations, No/Very minor concerns regarding coherence, No/Very minor concerns regarding adequacy, and Minor concerns regarding relevance | Lapierre et al. 2006; Alcock GA et al. 2009; Esienumoh EE et al. 2018; Higgins-Steele A et al. 2015; Rath S et al. 2010; |
| 10 | Tools aimed at assessing the knowledge of the other (35 -38) and promoting group participation were observed, such as: explaining the value of experience, collaborative planning processes, and participatory games. (35-38, 42). | Moderate confidence | Minor concerns regarding methodological limitations, No/Very minor concerns regarding coherence, No/Very minor concerns regarding adequacy, and Minor concerns regarding relevance | Lapierre et al. 2006; Alcock GA et al. 2009; Esienumoh EE et al. 2018; Higgins-Steele A et al. 2015; Rath S et al. 2010; |
| **KNOWLEDGE TRANSLATION STRATEGIES** | | | | |
| 11 | Critical consciousness, this result was reflected in the perspective of the group's capacity to improve their conditions (36-40, 42), the appropriation of the problems and the analysis that allows us to get to the root of these (36); added to the possibility of transforming the future of pregnant women and the communities where they live. (37). | Moderate confidence | Minor concerns regarding methodological limitations, No/Very minor concerns regarding coherence, No/Very minor concerns regarding adequacy, and Minor concerns regarding relevance | Lapierre et al. 2006; Esienumoh EE et al. 2018; Higgins-Steele A et al. 2015; Rath S et al. 2010; Joseph C, et.al. 2020; Sarmiento I, et.al. 2021 |
| 12 | The development of skills was identified to identify problems, improve the performance of responsibilities, strengthen empathic skills, respect for the other, and the development of organizational skills (37, 38, 42). | Moderate confidence | Minor concerns regarding methodological limitations, No/Very minor concerns regarding coherence, No/Very minor concerns regarding adequacy, and Minor concerns regarding relevance | Lapierre et al. 2006; Higgins-Steele A et al. 2015; Rath S et al. 2010; |
| 13 | There was evidence of a strengthening of the capacities of local decision-makers (38), as well as the empowerment of the community in the management of projects, the planning of courses and group sessions, the strengthening of learning (38, 40, 42) and greater confidence and autonomy in decision-making (38). | Moderate confidence | Minor concerns regarding methodological limitations, No/Very minor concerns regarding coherence, No/Very minor concerns regarding adequacy, and Minor concerns regarding relevance | Lapierre et al. 2006; Alcock GA et al. 2009; Rath S et al. 2010; Joseph C, et.al. 2020; Sarmiento I, et.al. 2021 |
