# Supplementary material for: Community-Based Knowledge Translation Strategies for Maternal, Neonatal, and Perinatal Outcomes: A Systematic Review of Quantitative and Qualitative Data
Source: Int J Public Health. 2023 Apr 20;68:1605239. doi: 10.3389/ijph.2023.1605239 (PMC10157638; doi:10.3389/ijph.2023.1605239)
Supplement: Supplementary file 4 [file DataSheet3.docx]

**Supplemental material 3. Search strategies**

| **Database:** Ovid MEDLINE(R) and Epub Ahead of Print, In-Process & Other Non-Indexed Citations and Daily 1946 to June 08, 2022  **Date:** 09.06.2022  **Hits:** 8154 |
| --- |
| # Searches Results  1 Community Health Workers/ 6258  2 Allied Health Personnel/ 12710  3 Voluntary Workers/ 10564  4 Doulas/ 191  5 (((lay or voluntary or volunteer? or untrained or unlicensed or nonprofessional? or non professional? or nonspecialist* or non specialist*) adj5 (worker? or visitor? or attendant? or aide or aides or support* or person* or helper? or career? or caregiver? or care giver? or consultant? or assistant? or staff or visit* or midwife or midwives)) or (traditional and (midwife or midwives))).ti,ab,kf. 12040  6 ((lay adj (volunteer* or health* or wom?n)) or laywom?n).ti,ab,kf. 1151  7 (paraprofessional? or para-professional? or paramedic or paramedics or paramedical worker? or paramedical personnel or allied health personnel or allied health worker? or support worker? or home health aide?).ti,ab,kf. 10390  8 (trained adj3 (volunteer? or health worker? or mother? or community member?)).ti,ab,kf. 2288  9 (((community or village?) adj3 (health worker? or health care worker? or healthcare worker?)) or community health assistant* or community based worker*).ti,ab,kf. 7372  10 (community adj3 (volunteer? or aide or aides)).ti,ab,kf. 2333  11 ((birth or childbirth or labor or labour) adj (attendant? or assistant?)).ti,ab,kf. 2636  12 (doula? or douladural?).ti,ab,kf. 468  13 monitrice?.ti,ab,kf. 8  14 (peer adj (volunteer? or counsel* or support or intervention?)).ti,ab,kf. 6853  15 (community based adj3 (approach* or intervention* or program* or counsel* or strateg*)).ti. 3382  16 (linkworker? or link worker?).ti,ab,kf. 123  17 barefoot doctor?.ti,ab,kf. 146  18 ((care or aide or aides or nursing or support or intervention? or treatment? or visit*) adj3 (lay or volunteer? or voluntary)).ti,ab,kf. 5301  19 1 or 2 or 3 or 4 or 5 or 6 or 7 or 8 or 9 or 10 or 11 or 12 or 13 or 14 or 15 or 16 or 17 or 18 68545  20 exp Pregnancy/ 970107  21 exp Pregnancy Complications/ 460372  22 exp Fetus/ 164377  23 exp Fetal Therapies/ 4415  24 exp Fetal Monitoring/ 9006  25 exp Prenatal Diagnosis/ 79324  26 exp Infant care/ or exp perinatal care/ 21225  27 Labor pain/ 1305  28 exp Obstetric Surgical Procedures/ 144017  29 Infant, Newborn/ 650531  30 exp Postpartum Period/ 72257  31 Breastfeeding/ 42031  32 (antenatal* or ante-natal* or prenatal* or pre-natal* or puerper* or postnatal* or post-natal* or postpartum or post-partum or perinatal* or peri-natal*).ti,ab,kf. 384687  33 (prepregnancy or pre-pregnancy or preconcept* or pre-concept* or periconcept* or peri-concept*).ti,ab,kf. 17743  34 ((preterm or pre-term or premature or pre-mature) and (labour or labor)).ti,ab,kf. 16375  35 (eclamp* or preeclamp*).ti,ab,kf. 41187  36 (breastfe* or breast-fe* or lactation).ti,ab,kf. 87122  37 (newborn* or new born*).ti,ab,kf. 194003  38 (pregnancy or pregnant or pregnancies).ti. 249260  39 exp Maternal Health Services/ 55731  40 (((maternal or maternity or motherhood) adj3 (service* or care)) or "maternal and child" or "maternal and infant" or (maternal adj (child or infant))).ti,ab,kf. 34176  41 20 or 21 or 22 or 23 or 24 or 25 or 26 or 27 or 28 or 29 or 30 or 31 or 32 or 33 or 34 or 35 or 36 or 37 or 38 or 39 or 40 1799518  42 19 and 41 8706  43 exp animals/ not humans.sh. 5015643  44 (news or editorial or comment).pt. 1577900  45 43 or 44 6534626  46 42 not 45 8154 |

| **Database:** [OVID] Embase 1974 to 2019 October 23  **Date:** 25.10.2019  **Hits:** 2747 |
| --- |
| # Searches Results  1 health auxiliary/ 6316  2 *paramedical personnel/ 6521  3 doula/ 200  4 traditional birth attendant/ 567  5 voluntary worker/ 5033  6 (((lay or voluntary or volunteer? or untrained or unlicensed or nonprofessional? or non professional? or nonspecialist* or non specialist*) adj5 (worker? or visitor? or attendant? or aide or aides or support* or person* or helper? or carer? or caregiver? or care giver? or consultant? or assistant? or staff or visit* or midwife or midwives)) or (traditional adj (midwife or midwives))).ti,ab,kw. 12183  7 ((lay adj (volunteer* or health* or wom?n)) or laywom?n).ti,ab,kw. 1129  8 (paraprofessional? or para-professional? or paramedic or paramedics or paramedical worker? or paramedical personnel or allied health personnel or allied health worker? or support worker? or home health aide?).ti,ab,kw. 11690  9 (trained adj3 (volunteer? or health worker? or mother? or community member?)).ti,ab,kw. 2498  10 (((community or village?) adj3 (health worker? or health care worker? or healthcare worker?)) or community health assistant* or community based worker*).ti,ab,kw. 6417  11 (community adj3 (volunteer? or aide or aides)).ti,ab,kw. 2444  12 ((birth or childbirth or labor or labour) adj (attendant? or assistant?)).ti,ab,kw. 2334  13 (doula? or douladural?).ti,ab,kw. 316  14 monitrice?.ti,ab,kw. 5  15 (peer adj (volunteer? or counsel* or support or intervention?)).ti,ab,kw. 6483  16 (community based adj3 (approach* or intervention* or program* or counsel* or strateg*)).ti. 3339  17 (linkworker? or link worker?).ti,ab,kw. 99  18 barefoot doctor?.ti,ab,kw. 134  19 ((care or aide or aides or nursing or support or intervention? or treatment? or visit*) adj3 (lay or volunteer? or voluntary)).ti,ab,kw. 6504  20 or/1-19 60914  21 exp *pregnancy/ 173396  22 exp *pregnancy complication/ 64518  23 exp *pregnancy disorder/ 290669  24 exp *fetus monitoring/ 6408  25 *fetal therapy/ 247  26 exp *prenatal diagnosis/ 48445  27 *infant care/ 287  28 *labor pain/ 1172  29 exp *obstetric procedure/ 194300  30 exp *infant/ 46803  31 exp *childbirth/ 22728  32 *breast feeding/ 22365  33 (antenatal* or ante-natal* or prenatal* or pre-natal* or puerper* or postnatal* or post-natal* or postpartum or post-partum or perinatal* or peri-natal*).ti,ab,kw. 423022  34 (prepregnancy or pre-pregnancy or preconcept* or pre-concept* or periconcept* or peri-concept*).ti,ab,kw. 20543  35 ((preterm or pre-term or premature or pre-mature) and (labour or labor)).ti,ab,kw. 21022  36 (eclamp* or preeclamp*).ti,ab,kw. 50517  37 (breastfe* or breast-fe* or lactation).ti,ab,kw. 85361  38 (newborn* or new born*).ti,ab,kw. 193395  39 (pregnancy or pregnant or pregnancies).ti. 244230  40 *maternal welfare/ 4947  41 *maternal health service/ 342  42 (((maternal or maternity or motherhood) adj3 (service* or care)) or "maternal and child" or "maternal and infant" or (maternal adj (child or infant))).ti,ab,kw. 28750  43 or/21-42 1125432  44 20 and 43 5775  45 (exp animals/ or exp invertebrate/ or animal experiment/ or animal model/ or animal tissue/ or animal cell/ or nonhuman/) not (human/ or normal human/ or human cell/) 6345209  46 (news or editorial or comment).pt. 636183  47 44 not (45 or 46) 5714  48 limit 47 to embase 2805  49 remove duplicates from 48 2747 |

| **Database:** [OVID] PsycINFO 1806 to October Week 2 2019  **Date:** 25.10.2019  **Hits:** 982 |
| --- |
| # Searches Results  1 allied health personnel/ 1035  2 community involvement/ 4472  3 volunteers/ 4604  4 (((lay or voluntary or volunteer? or untrained or unlicensed or nonprofessional? or non professional? or nonspecialist* or non specialist*) adj5 (worker? or visitor? or attendant? or aide or aides or support* or person* or helper? or carer? or caregiver? or care giver? or consultant? or assistant? or staff or visit* or midwife or midwives)) or (traditional adj (midwife or midwives))).ti,ab,id. 6338  5 ((lay adj (volunteer* or health* or wom?n)) or laywom?n).ti,ab,id. 441  6 (paraprofessional? or para-professional? or paramedic or paramedics or paramedical worker? or paramedical personnel or allied health personnel or allied health worker? or support worker? or home health aide?).ti,ab,id. 3869  7 (trained adj3 (volunteer? or health worker? or mother? or community member?)).ti,ab,id. 654  8 (((community or village?) adj3 (health worker? or health care worker? or healthcare worker?)) or community health assistant* or community based worker*).ti,ab,id. 1244  9 (community adj3 (volunteer? or aide or aides)).ti,ab,id. 1171  10 ((birth or childbirth or labor or labour) adj (attendant? or assistant?)).ti,ab,id. 243  11 (doula? or douladural?).ti,ab,id. 116  12 monitrice?.ti,ab,id. 0  13 (peer adj (volunteer? or counsel* or support or intervention?)).ti,ab,id. 4918  14 (community based adj3 (approach* or intervention* or program* or counsel* or strateg*)).ti. 1518  15 (linkworker? or link worker?).ti,ab,id. 35  16 barefoot doctor?.ti,ab,id. 8  17 ((care or aide or aides or nursing or support or intervention? or treatment? or visit*) adj3 (lay or volunteer? or voluntary)).ti,ab,id. 2339  18 or/1-17 28592  19 exp pregnancy/ 40494  20 exp obstetrical complications/ 1520  21 postpartum depression/ 4479  22 prenatal diagnosis/ 680  23 fetus/ 1952  24 exp "labor (childbirth)"/ 1285  25 breast feeding/ 3464  26 perinatal period/ 2624  27 postnatal period/ 4387  28 prenatal care/ 1763  29 (antenatal* or ante-natal* or prenatal* or pre-natal* or puerper* or postnatal* or post-natal* or postpartum or post-partum or perinatal* or peri-natal*).ti,ab,id. 54090  30 (prepregnancy or pre-pregnancy or preconcept* or pre-concept* or periconcept* or peri-concept*).ti,ab,id. 2934  31 ((preterm or pre-term or premature or pre-mature) and (labour or labor)).ti,ab,id. 461  32 (eclamp* or preeclamp*).ti,ab,id. 576  33 (breastfe* or breast-fe* or lactation).ti,ab,id. 7566  34 (newborn* or new born*).ti,ab,id. 11309  35 (pregnancy or pregnant or pregnancies).ti. 14646  36 (((maternal or maternity or motherhood) adj3 (service* or care)) or "maternal and child" or "maternal and infant" or (maternal adj (child or infant))).ti,ab,id. 7849  37 or/19-36 100130  38 18 and 37 982  39 remove duplicates from 38 982 |

| **Database:** Cochrane Library (Cochrane Database of Systematic Reviews, CENTRAL) [Wiley]  **Date:** 09.06.2022  **Hits:** 1251 |
| --- |
| ID Search Hits  #1 [mh ^"Community Health Workers"] 567  #2 [mh ^"Allied Health Personnel"] 302  #3 [mh ^"Voluntary Workers"] 328  #4 [mh ^Doulas] 9  #5 (((lay or voluntary or volunteer? or untrained or unlicensed or nonprofessional? or non NEXT professional? or nonspecialist* or non NEXT specialist*) NEAR/5 (worker? or visitor? or attendant? or aide or aides or support* or person* or helper? or carer? or caregiver? or care NEXT giver? or consultant? or assistant? or staff or visit* or midwife or midwives)) or (traditional NEXT (midwife or midwives))):ti,ab 2104  #6 ((lay NEXT (volunteer* or health* or wom?n)) or laywom?n):ti,ab 394  #7 (paraprofessional? or para NEXT professional? or paramedic or paramedics or paramedical NEXT worker? or paramedical NEXT personnel or allied NEXT health NEXT personnel or allied NEXT health NEXT worker? or support NEXT worker? or home NEXT health NEXT aide?):ti,ab 1427  #8 (trained NEAR/3 (volunteer? or health NEXT worker? or mother? or community NEXT member?)):ti,ab 808  #9 (((community or village?) NEAR/3 (health NEXT worker? or health NEXT care NEXT worker? or healthcare NEXT worker?)) or community NEXT health NEXT assistant* or community NEXT based NEXT worker*):ti,ab 1815  #10 (community NEAR/3 (volunteer? or aide or aides)):ti,ab 460  #11 ((birth or childbirth or labor or labour) NEXT (attendant? or assistant?)):ti,ab 244  #12 (doula? or douladural?):ti,ab 77  #13 monitrice?:ti,ab 1  #14 (peer NEXT (volunteer? or counsel* or support or intervention?)):ti,ab 1674  #15 (community NEXT based NEAR/3 (approach* or intervention* or program* or counsel* or strateg*)):ti 935  #16 (linkworker? or link NEXT worker?):ti,ab 19  #17 (barefoot NEXT doctor?):ti,ab 0  #18 ((care or aide or aides or nursing or support or intervention? or treatment? or visit*) NEAR/3 (lay or volunteer? or voluntary)):ti,ab 2694  #19 {or #1-#18} 11164  #20 [mh Pregnancy] 24542  #21 [mh "Pregnancy Complications"] 12424  #22 [mh Fetus] 1893  #23 [mh "Fetal Therapies"] 46  #24 [mh "Fetal Monitoring"] 375  #25 [mh "Prenatal Diagnosis"] 879  #26 [mh "Infant care"] 830  #27 [mh "perinatal care"] 618  #28 [mh ^"Labor pain"] 455  #29 [mh "Obstetric Surgical Procedures"] 7784  #30 [mh ^"Infant, Newborn"] 17330  #31 [mh "Postpartum Period"] 1885  #32 [mh ^Breastfeeding] 2075  #33 (antenatal* or ante NEXT natal* or prenatal* or pre NEXT natal* or puerper* or postnatal* or post NEXT natal* or postpartum or post NEXT partum or perinatal* or peri NEXT natal*):ti,ab 25716  #34 (prepregnancy or pre NEXT pregnancy or preconcept* or pre NEXT concept* or periconcept* or peri NEXT concept*):ti,ab 1486  #35 ((preterm or pre NEXT term or premature or pre NEXT mature) and (labour or labor)):ti,ab 2445  #36 (eclamp* or preeclamp*):ti,ab 3677  #37 (breastfe* or breast NEXT fe* or lactation):ti,ab 10753  #38 (newborn* or new born*):ti,ab 11455  #39 (pregnancy or pregnant or pregnancies):ti 17027  #40 [mh "Maternal Health Services"] 2523  #41 (((maternal or maternity or motherhood) NEAR/3 (service* or care)) or "maternal and child" or "maternal and infant" or (maternal NEXT (child or infant))):ti,ab 3111  #42 {or #20-#41} 78700  #43 #19 and #42 in Cochrane Reviews 66  #44 #19 and #42 in Trials 1318  #45 #43 or #44 1251#45 #43 or #44 1251 |

| **Database:** CINAHL [EBSCO]  **Date:** 25.10.2019  **Hits:** 1847 |
| --- |
| # Query Results  S1 TI ( paraprofessional# or para-professional# or paramedic or paramedics or paramedical W0 worker# or paramedical W0 personnel or allied W0 health W0 personnel or allied W0 health W0 worker# or support W0 worker# or home W0 health W0 aide#) ) OR AB ( paraprofessional# or para-professional# or paramedic or paramedics or paramedical W0 worker# or paramedical W0 personnel or allied W0 health W0 personnel or allied W0 health W0 worker# or support W0 worker# or home W0 health W0 aide#) ) OR SU ( paraprofessional# or para-professional# or paramedic or paramedics or paramedical W0 worker# or paramedical W0 personnel or allied W0 health W0 personnel or allied W0 health W0 worker# or support W0 worker# or home W0 health W0 aide#) ) 10,862  S2 TI ( antenatal* or ante-natal* prenatal* or pre-natal* or puerper* or postnatal* or post W0 natal* or postpartum or post W0 partum or perinatal* or peri W0 natal* ) OR AB ( antenatal* or ante-natal* prenatal* or pre-natal* or puerper* or postnatal* or post W0 natal* or postpartum or post W0 partum or perinatal* or peri W0 natal*antenatal* or ante-natal* prenatal* or pre-natal* or puerper* or postnatal* or post W0 natal* or postpartum or post W0 partum or perinatal* or peri W0 natal* ) OR SU ( antenatal* or ante-natal* prenatal* or pre-natal* or puerper* or postnatal* or post W0 natal* or postpartum or post W0 partum or perinatal* or peri W0 natal* ) 74,039  S3 TI ( ((preterm or pre-term or premature or pre-mature) and (labour or labor)) ) OR AB ( ((preterm or pre-term or premature or pre-mature) and (labour or labor)) ) OR SU ( ((preterm or pre-term or premature or pre-mature) and (labour or labor)) ) 5,387  S4 TI ( (((maternal or maternity or motherhood) N2 (service* or care)) or "maternal and child" or "maternal and infant" or (maternal W0 (child or infant))) ) OR AB ( (((maternal or maternity or motherhood) N2 (service* or care)) or "maternal and child" or "maternal and infant" or (maternal W0 (child or infant))) ) OR SU ( (((maternal or maternity or motherhood) N2 (service* or care)) or "maternal and child" or "maternal and infant" or (maternal W0 (child or infant))) ) 22,631  S5 (MH "Community Health Workers") 2,921  S6 (MH "Allied Health Personnel") 3,701  S7 (MH "Rural Health Personnel") 569  S8 (MH "Volunteer Workers") 12,544  S9 (MH "Doulas") 581  S10 TI ( (((lay or voluntary or volunteer# or untrained or unlicensed or nonprofessional# or non W0 professional# or nonspecialist* or non W0 specialist*) N4 (worker# or visitor# or attendant# or aide or aides or support* or person* or helper# or carer# or caregiver# or care W0 giver# or consultant# or assistant# or staff or visit* or midwife or midwives)) or (traditional W0 (midwife or midwives))) ) OR AB ( (((lay or voluntary or volunteer# or untrained or unlicensed or nonprofessional# or non W0 professional# or nonspecialist* or non W0 specialist*) N4 (worker# or visitor# or attendant# or aide or aides or support* or person* or helper# or carer# or caregiver# or care W0 giver# or consultant# or assistant# or staff or visit* or midwife or midwives)) or (traditional W0 (midwife or midwives))) ) OR SU ( (((lay or voluntary or volunteer# or untrained or unlicensed or nonprofessional# or non W0 professional# or nonspecialist* or non W0 specialist*) N4 (worker# or visitor# or attendant# or aide or aides or support* or person* or helper# or carer# or caregiver# or care W0 giver# or consultant# or assistant# or staff or visit* or midwife or midwives)) or (traditional W0 (midwife or midwives))) ) 19,764  S11 TI ( ((lay W0 (volunteer* or health* or wom#n)) or laywom#n) ) OR AB ( ((lay W0 (volunteer* or health* or wom#n)) or laywom#n) ) OR SU ( ((lay W0 (volunteer* or health* or wom#n)) or laywom#n) ) 530  S12 TI ( paraprofessional# or para-professional# or paramedic or paramedics or paramedical W0 worker# or paramedical W0 personnel or allied W0 health W0 personnel or allied W0 health W0 worker# or support W0 worker# or home W0 health W0 aide#) ) OR AB ( paraprofessional# or para-professional# or paramedic or paramedics or paramedical W0 worker# or paramedical W0 personnel or allied W0 health W0 personnel or allied W0 health W0 worker# or support W0 worker# or home W0 health W0 aide#) ) OR SU ( paraprofessional# or para-professional# or paramedic or paramedics or paramedical W0 worker# or paramedical W0 personnel or allied W0 health W0 personnel or allied W0 health W0 worker# or support W0 worker# or home W0 health W0 aide#) ) 10,862  S13 TI ( (trained N2 (volunteer# or health W0 worker# or mother# or community W0 member#)) ) OR AB ( (trained N2 (volunteer# or health W0 worker# or mother# or community W0 member#)) ) OR SU ( (trained N2 (volunteer# or health W0 worker# or mother# or community W0 member#)) ) 748  S14 TI ( (((community or village#) N2 (health W0 worker# or health W0 care W0 worker# or healthcare W0 worker#)) or community W0 health W0 assistant* or community W0 based W0 worker*) ) OR AB ( (((community or village#) N2 (health W0 worker# or health W0 care W0 worker# or healthcare W0 worker#)) or community W0 health W0 assistant* or community W0 based W0 worker*) ) OR SU ( (((community or village#) N2 (health W0 worker# or health W0 care W0 worker# or healthcare W0 worker#)) or community W0 health W0 assistant* or community W0 based W0 worker*) ) 4,273  S15 TI ( (community N2 (volunteer# or aide or aides)) ) OR AB ( (community N2 (volunteer# or aide or aides)) ) OR SU ( (community N2 (volunteer# or aide or aides)) ) 909  S16 TI ( ((birth or childbirth or labor or labour) W0 (attendant# or assistant#)) ) OR AB ( ((birth or childbirth or labor or labour) W0 (attendant# or assistant#)) ) OR SU ( ((birth or childbirth or labor or labour) W0 (attendant# or assistant#)) ) 984  S17 TI ( (doula# or douladural#) ) OR AB ( (doula# or douladural#) ) OR SU ( (doula# or douladural#) ) 721  S18 TI monitrice# OR AB monitrice# OR SU monitrice# 5  S19 TI ( (peer W0 (volunteer# or counsel* or support or intervention#)) ) OR AB ( (peer W0 (volunteer# or counsel* or support or intervention#)) ) OR SU ( (peer W0 (volunteer# or counsel* or support or intervention#)) ) 4,334  S20 TI ((community W0 based) N2 (approach* or intervention* or program* or counsel* or strateg*)) 1,956  S21 TI ( (linkworker# or link W0 worker#) ) OR AB ( (linkworker# or link W0 worker#) ) OR SU ( (linkworker# or link W0 worker#) ) 72  S22 TI Barefoot W0 doctor# OR AB Barefoot W0 doctor# OR SU Barefoot W0 doctor# 16  S23 TI ( ((care or aide or aides or nursing or support or intervention# or treatment# or visit*) N2 (lay or volunteer# or voluntary)) ) OR AB ( ((care or aide or aides or nursing or support or intervention# or treatment# or visit*) N2 (lay or volunteer# or voluntary)) ) OR SU ( ((care or aide or aides or nursing or support or intervention# or treatment# or visit*) N2 (lay or volunteer# or voluntary)) ) 2,308  S24 S5 OR S6 OR S7 OR S8 OR S9 OR S10 OR S11 OR S12 OR S13 OR S14 OR S15 OR S16 OR S17 OR S18 OR S19 OR S20 OR S21 OR S22 OR S23 43,617  S25 (MH "Pregnancy+") 185,737  S26 (MH "Pregnancy Complications+") 80,642  S27 (MH "Fetus+") 23,112  S28 (MH "Fetal Monitoring+") 2,686  S29 (MH "Prenatal Diagnosis+") 17,448  S30 (MH "Infant Care+") 5,035  S31 (MH "Labor Pain") 1,900  S32 (MH "Obstetric Care+") 44,266  S33 (MH "Infant, Newborn") 113,767  S34 (MH "Postnatal Period+") 11,535  S35 (MH "Breast Feeding") 20,358  S36 TI ( antenatal* or ante-natal* prenatal* or pre-natal* or puerper* or postnatal* or post W0 natal* or postpartum or post W0 partum or perinatal* or peri W0 natal* ) OR AB ( antenatal* or ante-natal* prenatal* or pre-natal* or puerper* or postnatal* or post W0 natal* or postpartum or post W0 partum or perinatal* or peri W0 natal*antenatal* or ante-natal* prenatal* or pre-natal* or puerper* or postnatal* or post W0 natal* or postpartum or post W0 partum or perinatal* or peri W0 natal* ) OR SU ( antenatal* or ante-natal* prenatal* or pre-natal* or puerper* or postnatal* or post W0 natal* or postpartum or post W0 partum or perinatal* or peri W0 natal* ) 74,039  S37 TI ( (prepregnancy or pre W0 pregnancy or preconcept* or pre W0 concept* or periconcept* or peri W0 concept*) ) OR AB ( (prepregnancy or pre W0 pregnancy or preconcept* or pre W0 concept* or periconcept* or peri W0 concept*) ) OR SU ( (prepregnancy or pre W0 pregnancy or preconcept* or pre W0 concept* or periconcept* or peri W0 concept*) ) 6,531  S38 TI ( ((preterm or pre-term or premature or pre-mature) and (labour or labor)) ) OR AB ( ((preterm or pre-term or premature or pre-mature) and (labour or labor)) ) OR SU ( ((preterm or pre-term or premature or pre-mature) and (labour or labor)) ) 5,387  S39 TI ( (eclamp* or preeclamp*) ) OR AB ( (eclamp* or preeclamp*) ) OR SU ( (eclamp* or preeclamp*) ) 12,103  S40 TI ( (breastfe* or breast W0 fe* or lactation) ) OR AB ( (breastfe* or breast W0 fe* or lactation) ) OR SU ( (breastfe* or breast W0 fe* or lactation) ) 30,185  S41 TI ( (newborn* or new W0 born*) ) OR AB ( (newborn* or new W0 born*) ) OR SU ( (newborn* or new W0 born*) ) 124,157  S42 TI (pregnancy or pregnant or pregnancies) 58,159  S43 TI ( (((maternal or maternity or motherhood) N2 (service* or care)) or "maternal and child" or "maternal and infant" or (maternal W0 (child or infant))) ) OR AB ( (((maternal or maternity or motherhood) N2 (service* or care)) or "maternal and child" or "maternal and infant" or (maternal W0 (child or infant))) ) OR SU ( (((maternal or maternity or motherhood) N2 (service* or care)) or "maternal and child" or "maternal and infant" or (maternal W0 (child or infant))) ) 22,631  S44 S25 OR S26 OR S27 OR S28 OR S29 OR S30 OR S31 OR S32 OR S33 OR S34 OR S35 OR S36 OR S37 OR S38 OR S39 OR S40 OR S41 OR S42 OR S43 347,767  S45 S24 AND S44 4,049  S46 S24 and S44 [Limiters - Exclude MEDLINE records] 1,847 |

| **Database:** Web of Science (SCI-EXPANDED, SSCI) [Clarivate]  **Date:** 09.06.2022  **Hits:** 4303 |
| --- |
| # 1 TOPIC: (((("lay" or "voluntary" or volunteer* or "untrained" or "unlicensed" or nonprofessional* or non-professional* or nonspecialist* or non-specialist*) NEAR/4 (worker* or visitor* or attendant* or "aide" or "aides" or support* or person* or helper* or carer* or caregiver* or care-giver* or consultant* or assistant* or "staff" or visit* or "midwife" or "midwives")) or ("traditional" NEAR/0 ("midwife" or "midwives")))) 10,992  # 2 TOPIC: ((("lay" NEAR/0 (volunteer* or health* or wom*n)) or laywom*n or paraprofessional* or para-professional* or "paramedic" or "paramedics" or paramedical-worker* or "paramedical personnel" or "allied health personnel" or allied-health-worker* or support-worker* or home-health-aide* or ("trained" NEAR/2 (volunteer* or health-worker* or mother* or community-member*)))) 10,759  # 3 TOPIC: (((("community" or village*) NEAR/2 (health-worker* or health-care-worker* or healthcare-worker*)) or community-health-assistant* or community-based-worker* or ("community" NEAR/2 (volunteer* or "aide" or "aides")) or birth-attendant* or birth-assistant* or childbirth-attendant* or childbirth-assistant* or labor-attendant* or labor-assistant* or labour-attendant* or labour-assistant* or doula* or douladural* or monitrice* or peer-volunteer* or peer-counsel* or peer-support or peer-intervention* or linkworker* or link-worker* or Barefoot-doctor* or (("care" or "aide" or "aides" or "nursing" or "support" or intervention* or treatment* or visit*) NEAR/2 ("lay" or volunteer* or "voluntary")))) 24,414  # 4 TITLE: ((("community based" NEAR/2 (approach* or intervention* or program* or counsel* or strateg*)))) 4,026  # 5 #4 OR #3 OR #2 OR #1 45,232  # 6 TOPIC: ((antenatal* or ante-natal* or prenatal* or pre-natal* or puerper* or postnatal* or post-natal* or "postpartum" or "post-partum" or perinatal* or peri-natal* or "prepregnancy" or "pre-pregnancy" or preconcept* or pre-concept* or periconcept* or peri-concept* or (("preterm" or "pre-term" or "premature" or "pre-mature") and ("labour" or "labor")) or eclamp* or preeclamp* or breastfe* or breast-fe* or "lactation" or newborn* or new-born* or neonate* or ((maternal or maternity or motherhood) N2 (service* or care)) or "maternal and child" or "maternal and infant" or (maternal W0 (child or infant)))) 528,927  # 7 TITLE: (("pregnancy" or "pregnant" or "pregnancies")) 166,316  # 8 #7 OR #6 633,668  # 9 #8 AND #5 [Indexes=SCI-EXPANDED, SSCI Timespan=1987-2022] 4,303 |

| **Database:** Epistemonikos  **Date:** 09.06.2022  **Hits:** 341 |
| --- |
| **SEARCH 1**: 4 Broad Synthesis, 3 Structured Summaries, 85 Systematic Reviews  [Title/Abstracts:]("lay health" OR "lay workers" OR "lay volunteer" OR "lay volunteers" OR "lay women" OR "lay woman" OR laywomen OR laywoman OR unlicensed OR untrained OR "voluntary worker" OR "voluntary workers" OR "voluntary health worker" OR "voluntary health workers" OR "voluntary healthcare worker" OR "voluntary healthcare workers" OR "voluntary health care worker" OR "voluntary health care workers" OR nonprofessional* OR non-professional* OR nonspecialist* OR non-specialist* OR "traditional midwife" OR "traditional midwives" OR paraprofessional* OR para-professional* OR paramedic OR paramedics OR "paramedical worker" OR "paramedical workers" OR "paramedical personnel" OR "allied health personnel" OR "allied health worker" OR "allied health workers" OR "support worker" OR "support workers" OR "home health aide" OR "home health aides" OR "trained mother" OR "trained mothers" OR "trained community" OR "community health worker" OR "community health workers" OR "community healthcare worker" OR "community healthcare workers" OR "community health care worker" OR "community health care workers" OR "community health assistant" OR "community health assistants" OR "community based worker" OR "community based workers")  AND  [Title/Abstracts:] (antenatal* OR ante-natal* or prenatal* OR pre-natal* OR puerper* OR postnatal* OR post-natal* OR "postpartum" OR "post-partum" OR perinatal* OR peri-natal* OR "prepregnancy" OR "pre-pregnancy" OR preconcept* OR pre-concept* OR periconcept* OR peri-concept* OR childbirth OR eclamp* OR preeclamp* OR breastfe* OR "breast-feeding" OR "lactation" OR newborn* OR "new-born" OR "new-borns" OR neonate* OR neo-nate* OR "maternal health services" OR "maternal healthcare services" OR "maternal-child" OR "maternity service" OR "maternity services" OR "motherhood service" OR "motherhood services" OR "maternal care" OR "maternal healthcare" OR "maternal health care" OR "pregnancy" OR "pregnant" OR "pregnancies")  **SEARCH 2**: 5 Broad Synthesis, 12 Structured Summaries, 112 Systematic Reviews  [Title/Abstracts:] ("birth attendant" OR "birth attendants" OR "birth assistant" OR "birth assistants" OR "childbirth attendant" OR "childbirth attendants" OR "childbirth assistant" OR "childbirth assistants" OR "labor attendant" OR "labor attendants" OR "labor assistant" OR "labor assistants" OR "labour attendant" OR "labour attendants" OR "labour assistant" OR "labour assistants" OR doula* OR douladural* OR monitrice* OR "peer counseling" OR "peer support" OR "peer intervention" OR "peer interventions" OR linkworker* OR "link worker" OR "link workers" OR "barefoot doctor" OR "barefoot doctors" OR "community based approach" OR "community based approaches" OR "community based intervention" OR "community based interventions" OR "community based programs" OR "community based programmes" OR "community based programme" OR "community based program" OR "community based strategy" OR "community based strategies" OR "community based counseling")  AND  [Title/Abstracts:] (antenatal* OR ante-natal* or prenatal* OR pre-natal* OR puerper* OR postnatal* OR post-natal* OR "postpartum" OR "post-partum" OR perinatal* OR peri-natal* OR "prepregnancy" OR "pre-pregnancy" OR preconcept* OR pre-concept* OR periconcept* OR peri-concept* OR childbirth OR eclamp* OR preeclamp* OR breastfe* OR "breast-feeding" OR "lactation" OR newborn* OR "new-born" OR "new-borns" OR neonate* OR neo-nate* OR "maternal health services" OR "maternal healthcare services" OR "maternal-child" OR "maternity service" OR "maternity services" OR "motherhood service" OR "motherhood services" OR "maternal care" OR "maternal healthcare" OR "maternal health care" OR "pregnancy" OR "pregnant" OR "pregnancies") |

| **Database:** LILACS  **Date:** 09/06/2022  **Hits:** 429 |
| --- |
| ("lay health" OR "lay workers" OR "lay volunteer" OR "lay volunteers" OR "lay women" OR "lay woman" OR laywomen OR laywoman OR unlicensed OR untrained OR "voluntary worker" OR "voluntary workers" OR "voluntary health worker" OR "voluntary health workers" OR "voluntary healthcare worker" OR "voluntary healthcare workers" OR "voluntary health care worker" OR "voluntary health care workers" OR nonprofessional$ OR non-professional$ OR nonspecialist$ OR non-specialist$ OR "traditional midwife" OR "traditional midwives" OR paraprofessional$ OR para-professional$ OR paramedic OR paramedics OR "paramedical worker" OR "paramedical workers" OR "paramedical personnel" OR "allied health personnel" OR "allied health worker" OR "allied health workers" OR "support worker" OR "support workers" OR "home health aide" OR "home health aides" OR "trained mother" OR "trained mothers" OR "trained community" OR "community health worker" OR "community health workers" OR "community healthcare worker" OR "community healthcare workers" OR "community health care worker" OR "community health care workers" OR "community health assistant" OR "community health assistants" OR "community based worker" OR "community based workers" OR "birth attendant" OR "birth attendants" OR "birth assistant" OR "birth assistants" OR "childbirth attendant" OR "childbirth attendants" OR "childbirth assistant" OR "childbirth assistants" OR "labor attendant" OR "labor attendants" OR "labor assistant" OR "labor assistants" OR "labour attendant" OR "labour attendants" OR "labour assistant" OR "labour assistants" OR doula$ OR douladural$ OR monitrice$ OR "peer counseling" OR "peer support" OR "peer intervention" OR "peer interventions" OR linkworker$ OR "link worker" OR "link workers" OR "barefoot doctor" OR "barefoot doctors" OR "community based approach" OR "community based approaches" OR "community based intervention" OR "community based interventions" OR "community based programs" OR "community based programmes" OR "community based programme" OR "community based program" OR "community based strategy" OR "community based strategies" OR "community based counseling")  AND  (antenatal$ OR ante-natal$ or prenatal$ OR pre-natal$ OR puerper$ OR postnatal$ OR post-natal$ OR "postpartum" OR "post-partum" OR perinatal$ OR peri-natal$ OR "prepregnancy" OR "pre-pregnancy" OR preconcept$ OR pre-concept$ OR periconcept$ OR peri-concept$ OR childbirth OR eclamp$ OR preeclamp$ OR breastfe$ OR "breast-feeding" OR "lactation" OR newborn$ OR "new-born" OR "new-borns" OR neonate$ OR neo-nate$ OR "maternal health services" OR "maternal healthcare services" OR "maternal-child" OR "maternity service" OR "maternity services" OR "motherhood service" OR "motherhood services" OR "maternal care" OR "maternal healthcare" OR "maternal health care" OR "pregnancy" OR "pregnant" OR "pregnancies") |
