# Supplementary material for: Community-Based Knowledge Translation Strategies for Maternal, Neonatal, and Perinatal Outcomes: A Systematic Review of Quantitative and Qualitative Data
Source: Int J Public Health. 2023 Apr 20;68:1605239. doi: 10.3389/ijph.2023.1605239 (PMC10157638; doi:10.3389/ijph.2023.1605239)
Supplement: Supplementary file 5 [file DataSheet5.docx]

Supplementary material 5. Summary of the quality of qualitative studies. Critical Appraisal Skills Programme (CASP)

CASP

1 2 3 4 5 6 7 8 9 10

| Esienumoh, 2018 |  |  |  |  |  |  |  |  |  |  |
| --- | --- | --- | --- | --- | --- | --- | --- | --- | --- | --- |
| Alcock, 2009 |  |  |  |  |  |  |  |  |  |  |
| Rath, 2010 |  |  |  |  |  |  |  |  |  |  |
| Higgins-Steele, 2015 |  |  |  |  |  |  |  |  |  |  |
| Lapierre, 2005 |  |  |  |  |  |  |  |  |  |  |
| Sarmiento, 2020 |  |  |  |  |  |  |  |  |  |  |
| Joseph, 2021 |  |  |  |  |  |  |  |  |  |  |

1. Was there a clear statement of the aims of the research?
2. Is a qualitative methodology appropriate?
3. Was the research design appropriate to address the aims of the research?
4. Was the recruitment strategy appropriate to the aims of the research?
5. Was the data collected in a way that addressed the research issue?
6. Has the relationship between researcher and participants been adequately considered?
7. Have ethical issues been taken into consideration?
8. Was the data analysis sufficiently rigorous?
9. Is there a clear statement of findings?
10. How valuable is the research?
