# Supplementary material for: Community-Based Knowledge Translation Strategies for Maternal, Neonatal, and Perinatal Outcomes: A Systematic Review of Quantitative and Qualitative Data
Source: Int J Public Health. 2023 Apr 20;68:1605239. doi: 10.3389/ijph.2023.1605239 (PMC10157638; doi:10.3389/ijph.2023.1605239)
Supplement: Supplementary file 6 [file DataSheet2.docx]

**Supplementary material 2. Outcome definitions**

**Extreme maternal morbidity**: A severe complication that occurs during pregnancy at the time of delivery or within 42 days of termination of pregnancy, but survival is possible with prompt and timely medical intervention.

**Maternal mortality**: The death of a woman while pregnant or within 42 days after termination of pregnancy regardless of duration and place of pregnancy, from any cause related to or aggravated by the pregnancy or its gestation, but not from accidental or incidental causes.

**Mothers’ satisfaction with caregiving:** The satisfaction perceived by pregnant women with their labor and birth experience. In addition, this satisfaction is related to women’s personal expectations, pain relief, and the support received from relatives and health professionals, as well as the quality of the relationship with the latter and the fulfilment of mothers’ expectations. The level of satisfaction can be measured through dedicated or generic questionnaires.

**Spontaneous vaginal delivery:** Vaginal delivery that is manually assisted with no use of instrumentation such as forceps or vacuum extraction.

**Cesarean delivery:** Surgical procedure in which a fetus is delivered through an incision in the mother’s abdomen and uterus.

**Instrumental vaginal delivery**: The use of medical instruments to facilitate the birth of a child.

**Intact perineum:** Absence of perineal lacerations as source of significant discomfort to many women.

**Maternal mental health disorders**: Defined as the occurrence of disorders that start during the perinatal period. They include minor and major depression; anxiety disorders; post-traumatic stress disorder; bipolar disorder; schizophrenia and postpartum psychosis.

**Initiation of breastfeeding**: Pre and postpartum breastfeeding support program that recommends exclusive breastfeeding up to 6 months and breastfeeding up to 2 years, accompanied by an adequate complementary diet.

**Neonatal mortality:** Death occurred within the period between birth and the first 28 days of life.

**Neonatal morbidity:** Any of the following morbidities defined according to a 5-minute Apgar score lower than or equal to seven, neonate admission to a neonatal intensive care unit, respiratory morbidity including hyaline membrane disease, transient tachypnea of the newborn, pneumothorax, aspiration syndromes, need of neonatal mechanic ventilation, need of CPAP therapy, and other comorbidities including jaundice/need of phototherapy, hypoglycemia, parenteral nutrition, early sepsis, seizures, and hypoxic-ischemic encephalopathy.

**Perinatal mortality:** Death occurred within the period from 22 completed weeks of pregnancy (154 days or a fetus with a weight of 500g or more) to 7 days after birth.

**Community impact:** Considering that qualitative studies will be considered in this paper, social and/or community outcomes that could be identified through critical analysis of the results, such as: Recognition of social knowledge; network creation; recognition of social and political agency; revitalization of culture and traditional knowledge; strengthening of the community.
